# RNase-mediated protein footprint sequencing reveals protein-binding sites throughout the human transcriptome

**DOI:** 10.1186/gb-2014-15-1-r3

**Published:** 2014-01-07

**Authors:** Ian M Silverman, Fan Li, Anissa Alexander, Loyal Goff, Cole Trapnell, John L Rinn, Brian D Gregory

**Affiliations:** 1 Department of Biology, University of Pennsylvania, 433 S. University Ave, Philadelphia, PA 19104, USA; 2 PENN Genome Frontiers Institute, University of Pennsylvania, Philadelphia, PA 19104, USA; 3 Cell and Molecular Biology Graduate Group, University of Pennsylvania, Philadelphia, PA 19104, USA; 4 Genomics and Computational Biology Graduate Group, University of Pennsylvania, Philadelphia, PA 19104, USA; 5 Department of Stem Cell and Regenerative Biology, Harvard University, Cambridge, MA 02138, USA; 6 Broad Institute, Cambridge, MA 02142, USA; 7 Beth Israel Deaconess Medical Center, Boston, MA 02215, USA

## Abstract

Although numerous approaches have been developed to map RNA-binding sites of individual RNA-binding proteins (RBPs), few methods exist that allow assessment of global RBP–RNA interactions. Here, we describe PIP-seq, a universal, high-throughput, ribonuclease-mediated protein footprint sequencing approach that reveals RNA-protein interaction sites throughout a transcriptome of interest. We apply PIP-seq to the HeLa transcriptome and compare binding sites found using different cross-linkers and ribonucleases. From this analysis, we identify numerous putative RBP-binding motifs, reveal novel insights into co-binding by RBPs, and uncover a significant enrichment for disease-associated polymorphisms within RBP interaction sites.

## Background

RNA–protein interactions are central to all of the post-transcriptional regulatory processes that control gene expression. From the initial processing of a protein-coding transcript in the nucleus to its final translation and decay in the cytoplasm, cellular mRNAs are involved in a complex choreography with various transacting RNA-binding proteins (RBPs) [1-3]. RBPs are also required for the processing and function of the thousands of non-coding RNAs (ncRNAs), both large and small, encoded by eukaryotic genomes. These RNAs have a variety of cellular functions, including chromatin regulation and control of cell fate [4,5]. Thus, RNA–protein interactions represent a vast, diverse and critical layer of transcriptome regulation.

Eukaryotic genomes encode a large collection of RBPs that interact with mRNAs to form dynamic multi-component ribonucleoprotein complexes (mRNPs) [6,7]. These mRNPs often constitute the functional forms of mRNAs, and it is only through their proper formation that transcripts are correctly regulated to produce the precise required amounts of each protein in a cell [2,3,7,8]. Intriguingly, recent evidence suggests that post-transcriptional regulation of mRNAs encoding functionally related proteins likely requires mRNP assembly by specific sets of co-occurring RBPs, an idea that was originally postulated by the post-transcriptional operon hypothesis [9,10]. Thus, the precise composition and formation of RNPs in eukaryotic cells is critical for proper gene expression regulation.

The essential nature of RNA–protein interactions in eukaryotic biology has led to numerous biochemical, genetic and computational approaches being utilized, alone and in combination, to identify and validate RBPs and their specific RNA-binding sites [1,11,12]. These approaches have proven useful in characterizing a number of RBPs [13-26]. However, all of these earlier approaches investigated RNA–protein interactions one protein at a time, which limited their ability to monitor the global landscape of RNPs and reveal insights into the combinatorial binding and regulation by the cellular milieu of RBPs. Thus, there is a major gap between the significance of cellular RNA–RBP interactions and the difficulty in establishing a comprehensive catalogue of these interactions in a single experiment.

Recently, several groups have established experimental approaches for interrogating RNA–protein interaction sites on a more global scale. These approaches utilize 4-thiouridine and UV cross-linking to identify RNA–protein interactions by uncovering sites of T > C transversion (representing RNA–protein cross-linking events) [27,28]. However, these studies have been limited by several factors. Specifically, they rely on treatment with synthetic nucleotides and UV cross-linking, which can be used for cell cultures but not tissues or whole organisms. Furthermore, UV cross-linking only identifies sites of direct RNA–protein contact and may not capture the larger multi-protein complexes that make up the overall RNP architecture *in vivo*. Finally, these studies have focused on poly-adenylated (polyA) transcripts, reducing their ability to monitor RBP binding in non-polyA and nascent RNAs.

To address the limitations of the currently available methodologies, we present a ribonuclease (RNase)-mediated protein footprint sequencing approach that we call protein interaction profile sequencing (PIP-seq). This approach identifies RNA–protein interaction sites within both unprocessed and mature RNAs in a mostly unbiased manner and on a transcriptome-wide scale. We describe multiple cross-linking techniques to capture both direct and indirect RNA–protein interactions. We also show that both single-stranded and double-stranded RNases uncover distinct but overlapping sets of RNA–protein interaction sites. Using this approach, we find PIP-seq to be a reproducible approach that reveals both previously known and novel RBP interaction sites. We demonstrate the utility of PIP-seq by uncovering enriched sequence motifs within the complement of identified RBP interaction sites. We also investigate the interactions among protein-binding sites and provide evidence for co-binding of RNAs by specific sets of RBPs, some of which bind to groups of transcripts encoding functionally related proteins. These results reveal novel insights into networks of post-transcriptional gene regulation mediated by specific groups of RBP-bound sequence motifs. Finally, we identify a significant enrichment for disease-associated variants within RBP interaction sites, and demonstrate the effects of some of these single nucleotide polymorphisms (SNPs) on RNA–protein interactions. Overall, our approach provides an RNA-centric global assessment of RNA–RBP interactions that directly identifies RNA–protein interaction sites and is applicable for all organisms and sample types.

## Results and discussion

### An RNase-mediated protein footprint sequencing approach that identifies sites of RNA–protein interaction

**Figure 1:**
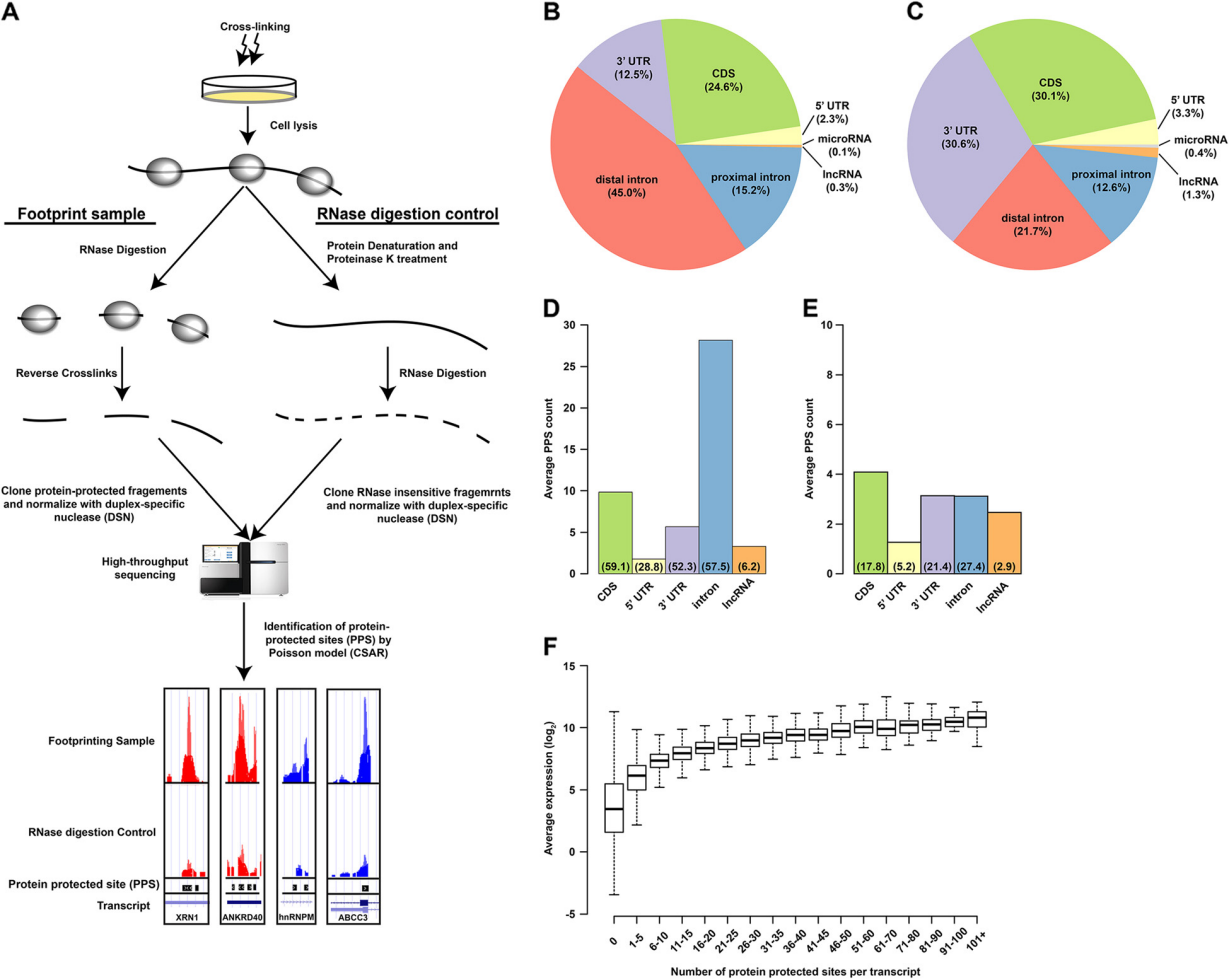
**Overview of the PIP-seq method. (A)** In the PIP-seq method, cells are cross-linked with formaldehyde or 254-nm UV light, or not cross-linked. They are lysed and divided into footprint and RNase digestion control samples. The footprint sample is treated with an RNase (ss- or dsRNase), which results in a population of RNase-protected RNA–RBP complexes. The protein cross-links are then reversed (by heating for formaldehyde cross-links or by proteinase K treatment for UV cross-links), leaving only the footprints where the RNA was protein-bound. For the RNase digestion control sample, which is designed to control for RNase insensitive regions, the order of operations is reversed; bound proteins are first removed by treatment with SDS and proteinase K, and then the unprotected RNA sample is subjected to RNase treatment. Strand-specific high-throughput sequencing libraries are prepared from both footprint and RNase digestion control samples and normalized using rehybridization and duplex-specific nuclease (DSN) treatment. PPSs are identified from the sequencing data using a Poisson model. Screenshots show UCSC browser views of sequencing reads from the footprint and RNase digestion control sample (same scale) and PPSs identified from the regions of the genes listed. **(B,C)** Absolute distribution of PPSs throughout RNA species for formaldehyde **(B)** and UV **(C)** cross-linked PIP-seq experiments. **(D,E)** Average PPS count per RNA molecule (classified by RNA type (mRNA and lncRNA) and transcript region (for example, 5′ UTR)) for formaldehyde **(D)** and UV **(E)** cross-linked PIP-seq experiments. Percentages indicate the fraction of each RNA type or region that contains PPS information. **(F)** Average expression (*y*-axis) of human mRNAs separated by total number of PPSs identified in their sequence (*x*-axis) for PPSs identified using formaldehyde cross-linking. CDS, coding sequence; DSN, duplex-specific nuclease; dsRNase, double-stranded RNase; lncRNA, long non-coding RNA; PIP-seq, protein interaction profile sequencing; PPS, protein-protected site; ssRNase, single-stranded RNase; UTR, untranslated region.

To obtain an unbiased, genome-wide view of RNA–protein interactions for both unprocessed and mature RNAs in eukaryotic transcriptomes, we developed an RNase-mediated protein footprint sequencing approach, known as PIP-seq, by performing nuclease-sensitivity sequencing assays [29,30] on cross-linked RNA–protein complexes from HeLa cells (Figure 1A). Previous investigations of RNA–protein interactions have assayed stable endogenous interactions as well as those captured by UV (254 nm), which cross-links only direct protein–nucleic acid contacts, and formaldehyde, which cross-links protein–nucleic acid and protein–protein contacts with a longer range [31-33]. Therefore, to generate a comprehensive and multifaceted view of RBP interaction sites, we used both cross-linking techniques and no cross-linking when performing PIP-seq.

We had previously used nuclease-sensitivity sequencing assays on purified RNAs to determine RNA base-pairing probabilities by treating RNA with either single-stranded or double-stranded RNase (ss- or dsRNase, respectively) and sequencing the resulting populations [29,30]. We reasoned that by using both of these RNases on cross-linked RNA–protein complexes, we would be able both to map RBP-binding sites comprehensively and also to investigate RNA base-pairing probabilities *in vivo.* However, for the purposes of this manuscript we focus our analysis specifically on the identification of protein-interaction sites, which we refer to as protein-protected sites (PPSs).

To perform PIP-seq, we started with adherent HeLa cells cross-linked by one of the methods described above (UV or formaldehyde) or used cells that had not been cross-linked. The resulting cell lysates were then split into experimental and background samples. Due to the structure-specific nature of the RNases used, it was essential to have a background sample to control for RNase insensitive regions. Therefore, a ‘footprint sample’ (experimental) was directly treated with either a single-stranded RNase (ssRNase), known as RNaseONE, or double-stranded RNase (dsRNase), known as RNaseV1. In contrast, the RNase digestion control sample was first denatured in SDS and treated with proteinase K prior to RNase digestion. In this way, regions that were protein-protected in the footprint sample became sensitive to RNase digestion in the control sample and regions that were unbound but insensitive to one of the nucleases due to their structural status, remained that way. For both samples, cross-links were subsequently reversed (by heating for formaldehyde cross-links or by extensive proteinase K treatment for UV cross-links), which was followed by strand-specific library preparation (Figure 1A). Highly abundant RNA species (for example, ribosomal RNAs) were depleted from each library based on their rapid re-annealing rates using a thermostable duplex-specific nuclease (DSN) protocol (see Materials and methods for more details).

We then sequenced the resulting libraries (four in total for each replicate) using the Illumina 50-bp single-end sequencing protocol, and obtained approximately 31 to 60 million raw reads per library (Additional file 1). To identify PPSs, we used a Poisson distribution model based on a modified version of the CSAR software package [34]. Specifically, the read coverage was calculated for each base position in the genome and a Poisson test was used to compute an enrichment score for the footprint versus RNase digestion control libraries (Additional file 1). PPSs were then called as described for ChIP-seq analysis [34] with a false discovery rate (FDR) of 5% (Figure 1A; for more examples see Additional file 2A to E). Using this approach we identified a total of approximately 1,011,000 PPSs over seven experiments, comprising approximately 430,000 non-overlapping sites (Additional file 1). Of note, saturation analysis indicated near linear growth in the number of PPSs relative to read depth, suggesting that further sequencing would likely uncover more PPSs, but with diminishing returns (Additional file 2F).

We found PPSs identified by both cross-linking strategies and with no cross-linking to be widely distributed across both exonic and intronic regions, with a particular enrichment for distal intronic binding in the formaldehyde-cross-linked experiments (Figure 1B,C and Additional file 3A). Closer examination of PPSs broken down by genic features (for example, 5′ and 3′ UTRs, coding sequence (CDS) and intron) or RNA type (mRNA and long non-coding RNA (lncRNA)) revealed that >50% of all human mRNAs contained multiple binding events across all transcript regions except 5′ UTR (average of approximately 1 PPS in only 28.8% of total transcripts) in HeLa cells (Figure 1D,E and Additional file 3B). Strikingly, an average of approximately 26 PPSs was found in the introns of each transcript in the formaldehyde-cross-linked PIP-seq experiments, compared with approximately three and approximately two intronic PPSs with the UV-cross-linked and non-cross-linked experiments, respectively (Figure 1D,E and Additional file 3B). These results suggest that formaldehyde cross-linking captures more transient and/or weak RBP–RNA interactions within intronic, especially distal (>500 nucleotides from a splice site), portions of mRNAs. We also found that approximately 2% to 6% of all known human lncRNAs could be identified as containing an average of 2.5 PPSs in HeLa cells using PIP-seq with the various cross-linking strategies (Figure 1D,E and Additional file 3B). The limited number of PPS-containing lncRNAs uncovered by our experiments is likely due to the low expression and tissue-specific nature of these transcripts. To address a possible dependence of our approach on RNA expression levels, we assessed the relation between RNA steady-state abundance and the number of PPSs per transcript and found that RNA levels explained only a small fraction (*R*^2^ = 0.11) of the total variation in PPS counts between transcripts (Figure 1F and Additional file 3C,D). Overall, these results suggest that PIP-seq provides a comprehensive and mostly unbiased view of global RNA–protein interaction sites in eukaryotic transcriptomes.

In general, we found that formaldehyde cross-linking revealed the highest number of PPSs, whereas UV and no cross-linking yielded many fewer sites (Additional file 1). This is not surprising, given that formaldehyde both has a longer range than UV and can also stabilize more transient and indirect interactions. Thus, the use of formaldehyde cross-linking gives a more comprehensive view of RNA–protein interaction sites, while the use of UV likely increases the specificity of PPSs to more tightly associated RBP-bound targets. We also observed that ssRNase treatment yielded twice as many unique PPSs compared to dsRNase digestion (Additional file 1). There are several explanations for this, none of which are mutually exclusive. For example, the ssRNase may have higher activity in the reaction conditions used in our experiments, the dsRNase may have lower accessibility to protein-bound dsRNA regions, or human RBPs may prefer non-structured regions within target RNAs for interaction. Together, these results show that the choice of cross-linking reagent or RNase can have a profound effect on RNA–protein interaction site identification and that these effects likely apply to the other technologies that address this same experimental question [27,28].

### PIP-seq is a reproducible approach for identifying known and novel RBP interaction sites

**Figure 2:**
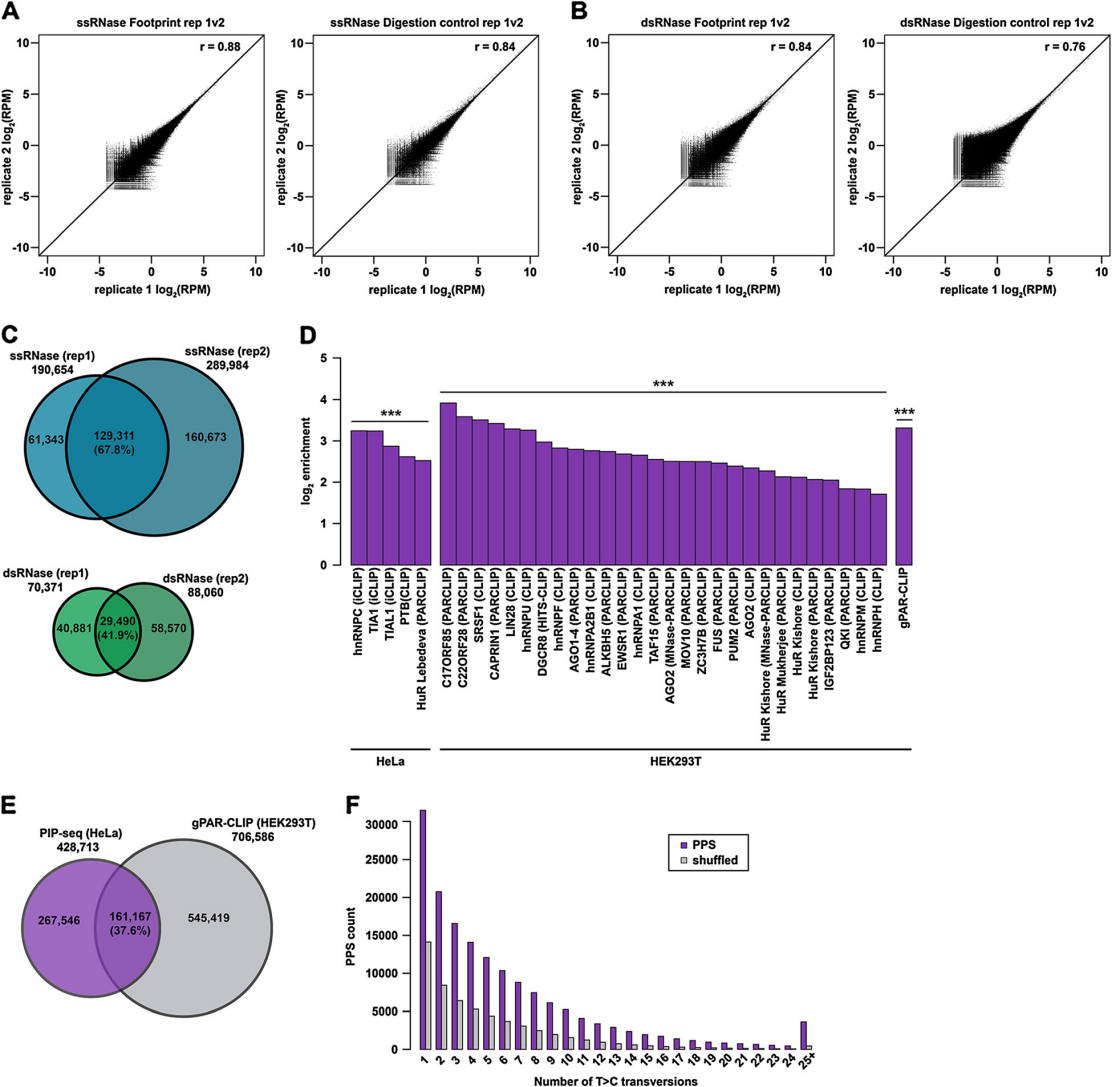
**PIP-seq is reproducible and captures known RBP–RNA interactions. (A)** Correlation in read counts between two formaldehyde-cross-linked ssRNase-treated PIP-seq replicates (footprint sample on left, RNase digestion control on right). **(B)** As **(A)**, but for formaldehyde-cross-linked dsRNase-treated replicates. **(C)** Overlap in PPS calls between formaldehyde-cross-linked ssRNase-treated (top, blue), and formaldehyde-cross-linked dsRNase-treated (bottom, green) PIP-seq replicates. **(D)** Overlap between PPSs identified from three formaldehyde-treated PIP-seq samples and various CLIP datasets. Values are shown as log_2_ enrichment over shuffled background distributions. *** denotes *P* < 2.2 × 10^-16^ (chi-squared test). **(E)** Overlap between formaldehyde-cross-linked PPSs from HeLa cells and 40-nucleotide T > C transversion event-containing loci from the gPAR-CLIP dataset generated from HEK293T cells (T > C transversion events less than 40 bp apart were merged to generate a dataset comparable to PPSs). **(F)** Number of T > C transversion events per PPS identified by formaldehyde cross-linking (purple) versus shuffled regions (gray). Values for the number of events per shuffled region are the average from ten random shuffles. bp, base pair; dsRNase, double-stranded RNase; PIP-seq, protein interaction profile sequencing; PPS, protein-protected site; ssRNase, single-stranded RNase.

To assess the reproducibility of PIP-seq, we first determined the correlation of sequencing read abundance between biological replicates of footprint and RNase digestion control libraries (Figure 2A,B and Additional file 4). Using a sliding-window approach, we observed a high correlation in read counts between individual replicates of formaldehyde-cross-linked ssRNase-treated footprint and RNase digestion control libraries (Pearson correlation *r* = 0.88 and 0.84, respectively) (Figure 2A and Additional file 4A,B). Similar results were also found for the dsRNase-treated libraries (Pearson correlation *r* = 0.84 and 0.76, footprint and RNase digestion control, respectively) (Figure 2B and Additional file 4A,B). This high reproducibility of PIP-seq libraries was also observed between replicates of the UV-cross-linked libraries (Additional file 4C). Together, these data indicate that PIP-seq experiments and controls are reproducible across replicates using various RNases and cross-linkers.

We next investigated the reproducibility of exact PPS identification between paired biological replicates. With formaldehyde cross-linking, we observed a 68% and 42% (for ssRNase and dsRNase, respectively) overlap between PPSs identified in two replicates (Figure 2C and Additional file 5A). Similarly, 73% and 64% (ssRNase and dsRNase, respectively) of the PPSs identified by UV cross-linking were replicated in a second larger dataset (Additional file 5B). This degree of overlap between PPSs is relatively high when compared to the more modest reproducibility of the identified RBP-binding sites in cross-linking and immunoprecipitation sequencing (CLIP-seq) and photoactivatable ribonucleoside cross-linking and immunoprecipitation (PAR-CLIP) experiments [18]. In total, these results indicate that our novel approach is a reproducible means of identifying the protein-bound component of the eukaryotic transcriptome.

We also interrogated the relation between PPSs identified by different RNases. We compared RNaseONE, which preferentially cleaves single-stranded RNA, to RNaseV1, which preferentially cleaves paired bases (Additional file 5C,D,E). We found a high overlap between formaldehyde-cross-linked PPSs (72%) identified by each RNase, compared to UV-cross-linked (32%) or non-cross-linked (37%) PPSs (Additional file 5C,D,E). This is unsurprising, given the larger number (Additional file 1) of PPSs identified using formaldehyde cross-linking compared to UV-cross-linked or non-cross-linked experiments. In total, these results revealed that both RNases uncovered a set of overlapping and unique PPS sequences, demonstrating that an ss- and dsRNase are needed for comprehensive identification of RNA–protein interaction sites in eukaryotic transcriptomes.

To validate that PIP-seq identifies bona fide RNA–protein interaction sites, we overlapped PPSs with known RBP-binding sites from HeLa and HEK293T cells [14-27], and found that a significant number (for most *P* < 2.2 × 10^–16^ – the exception is one HuR dataset for UV-cross-linked PPSs; see Additional file 6A) of the PPSs coincided with numerous RPB interaction sites previously tested by single protein immunoprecipitation approaches (for example, HITS-CLIP, PAR-CLIP and so on) compared to an expressed transcriptome background (see Materials and methods for more details) (Figure 2D and Additional file 6A,B). This is noteworthy given our analysis of PPSs in HeLa cells, since the majority of the CLIP-seq and PAR-CLIP datasets were generated using HEK293T cells.

We also compared our data with previously published global PAR-CLIP (gPAR-CLIP) data from HEK293T cells [27], in which protein-binding sites were identified on the basis of T > C transversions (Figure 2D,E and Additional file 6A,B,C,D). We observed a significant (*P* < 2.2 × 10^–16^) enrichment of the previously identified transversion events within our identified PPSs relative to the expressed transcriptome background, suggesting that at least some fraction of binding events are cell-type independent (there was an approximately 38% overlap between HeLa and HEK293T cells, Figure 2D,E and Additional file 6A,B,C,D). Furthermore, we analyzed the number of T > C transversions per PPS and found that on average 6.3 T > C transversions were observed per PPS for the formaldehyde-cross-linked PPSs (Figure 2F and Additional file 6E,F). These data revealed that there are often numerous gPAR-CLIP T > C transversions per RNA–protein-binding event identified by PIP-seq, and suggest that many of our identified PPSs are sites of multi-RNA-binding domain (RBD) and/or multi-RBP interactions. Additionally, our findings demonstrate that PIP-seq can identify the full footprint of RBP–RNA interaction sites, underscoring its utility in studying these events.

It is also worth noting that PIP-seq identified a total of 428,713 of approximately 40-nucleotide-long protein-protected regions, while gPAR-CLIP yielded 706,586 loci of similar length (Figure 2E). There are multiple explanations for this discrepancy. For instance, PIP-seq uses a background control library (RNase digestion control (Figure 1A)) whereas gPAR-CLIP does not. This control is likely important for distinguishing between noise and true protein-binding events, and may account for the identification of fewer sites by PIP-seq. Alternatively, PIP-seq may be less sensitive due to the lack of a stringent RNA–protein purification step. In total, our results indicate that PIP-seq captures a significant population of human RNA–protein interaction regions in a single experiment, further validating its reliability and robustness.

### PIP-seq gives an in-depth view of the protein-bound transcriptome

**Figure 3:**
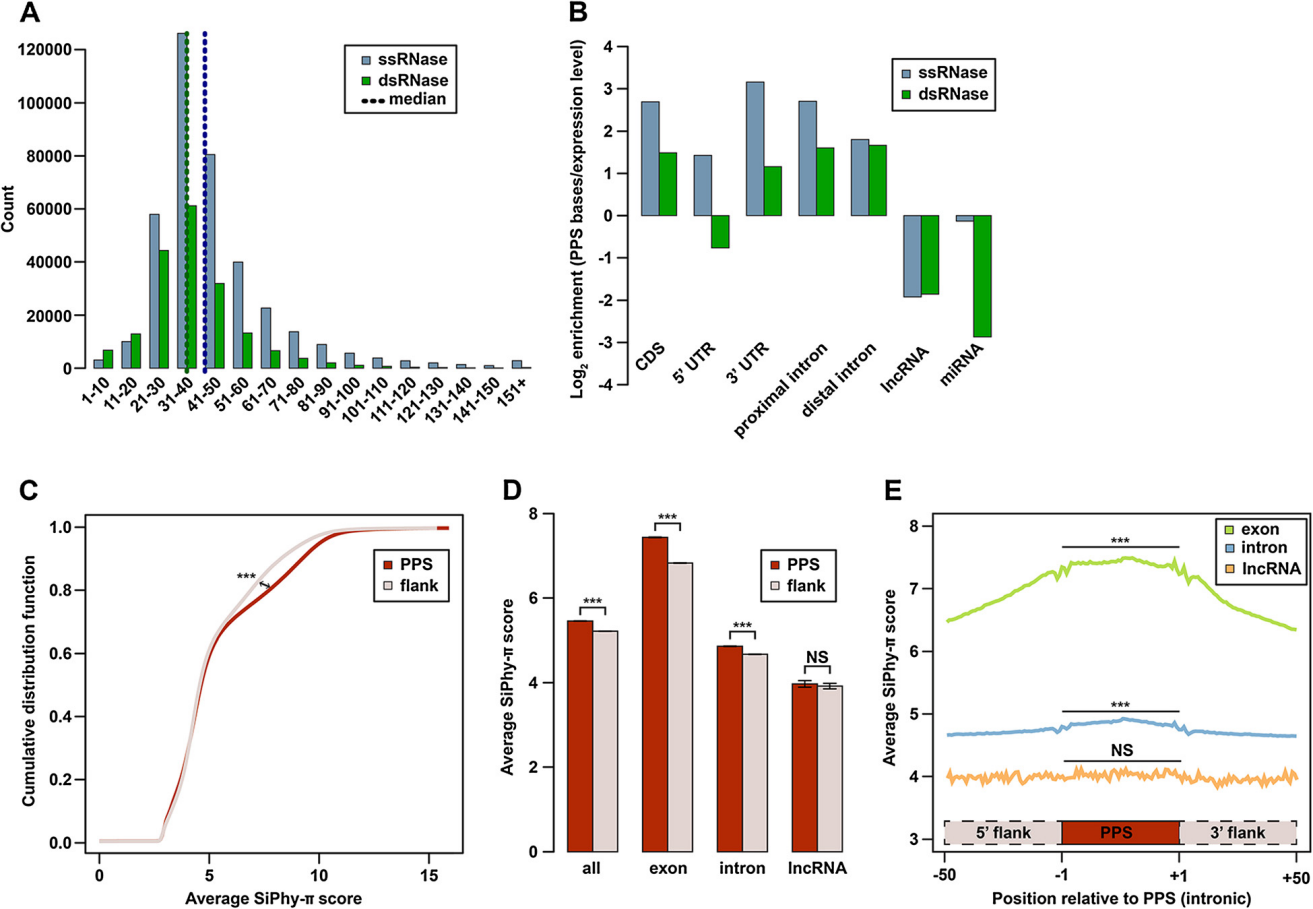
**Functional analysis and characterization of protein-binding sites. (A)** Distribution of ssRNase-treated (light blue bars) and dsRNase-treated (green bars) PPS sizes from formaldehyde-cross-linked samples. Dashed lines represent median PPS sizes (ssRNase, blue line and dsRNase, green line). **(B)** Genomic distribution of PPS density, measured as PPS base coverage normalized to RNase digestion control read counts per genomic region. Proximal intron refers to 500 nucleotides at the 5′ and 3′ ends of introns. **(C)** Cumulative distribution of average SiPhy-π scores in PPSs (red line) versus similarly sized flanking sequences (gray line). **(D)** Comparison of average SiPhy-π scores between PPSs (red bars) and flanking sequences (gray bars) for various genomic regions. **(E)** Average SiPhy-π score profiles across the first and last 25 nucleotides of PPSs as well as 50 nucleotides upstream and downstream of exonic (green line), intronic (blue line) and lncRNA (orange line) PPSs. *** denotes *P* < 2.2 × 10^–16^ (chi-squared test). CDS, coding sequence; dsRNase, double-stranded RNase; lncRNA, long non-coding RNA; NS, not significant; PPS, protein-protected site; ssRNase, single-stranded RNase; UTR, untranslated region.

Two outstanding questions in RNA biology are the extent and patterning of RBP binding across genic regions. We set out to address these questions using PIP-seq data from the various cross-linkers and RNases. We first determined the size distribution of PPSs identified using each RNase and cross-linker (Figure 3A). We found that the median PPS sizes for formaldehyde-cross-linked ss- and dsRNase treatments were approximately 40 and approximately 35 nucleotides, respectively. Importantly, this variation in size between the two RNases was consistent across cross-linkers (Additional file 7A,B), suggesting that ssRNase treatment reveals larger protein footprints and/or longer stretches of RBP interactions across RNA regions.

To assess the genomic distribution of protein-binding events, we calculated the enrichment of PPSs in specific regions of the human transcriptome (for example, CDS, 5′ UTR, 3′ UTR, intron and so on) relative to their expression levels in the RNase digestion control sample (Figure 3B and Additional file 7C,D). This analysis revealed a consistent enrichment between RNases and cross-linkers for protein-binding in the 3′ UTR, proximal (<500 nucleotides from a splice site) introns, as well as within the CDS (Figure 3B and Additional file 7C,D). These results are unsurprising given the role of these regions in post-transcriptional regulation and translation. We also found that distal (>500 nucleotides from a splice site) intronic regions were enriched for protein binding in the formaldehyde-treated samples only (Figure 3B), suggesting a high level of transient, weak and/or non-specific RNA-binding activity occurs in these non-coding areas. Our results support the idea that the large interior regions of introns may serve as sinks for RBPs in human cells [19].

In contrast to protein-coding mRNAs, we found that lncRNAs were consistently depleted for protein binding (Figure 3B and Additional file 7C,D). Therefore, we closely examined protein binding to the 100 most highly expressed lncRNAs compared to expression-matched mRNA 3′ UTRs in the three different cross-linking conditions. These analyses revealed that the fraction of identified lncRNA and 3′ UTR base pairs bound by proteins was similar for the formaldehyde cross-linking experiments using both RNases. Conversely, for UV and no cross-linking, lncRNAs were significant depleted in protein binding compared to the expression-matched mRNA 3′ UTRs (Additional file 7E). This depletion was consistent for both RNases, suggesting that this finding is not a consequence of structural differences between mRNAs and lncRNAs. In total, these results support the hypothesis that lncRNAs are more weakly and/or transiently bound by interacting proteins compared to protein-coding mRNAs, which may be a distinguishing feature of these two types of eukaryotic RNAs.

Given the fundamental role of RBP–RNA interactions in the regulation of eukaryotic gene expression, we hypothesized that many of the identified PPSs are evolutionarily conserved within vertebrates. To test this, we compared SiPhy-π conservation scores for PPSs versus same-sized neighboring regions (Figure 3C,D,E, and Additional file 8). Using this approach, we found that PPS sequences were significantly (*P* < 2.2 × 10^–16^) more evolutionarily conserved than flanking regions (Figure 3C and Additional file 8A,B). Importantly, this was true for PPS sequences in both exonic and intronic portions of human mRNAs, but not for lncRNAs (Figure 3D,E), and was consistent for PPSs identified with every cross-linking approach (Figure 3D,E and Additional file 8C,D,E,F). These results support the notion that the ability to interact with RBPs is functionally important to mRNA sequences, and that this trait has undergone selection during vertebrate evolution. Furthermore, the lack of conservation of PPSs within lncRNAs is consistent with their low conservation rates across vertebrate species.

### RBP-binding densities across unprocessed and mature mRNAs

**Figure 4:**
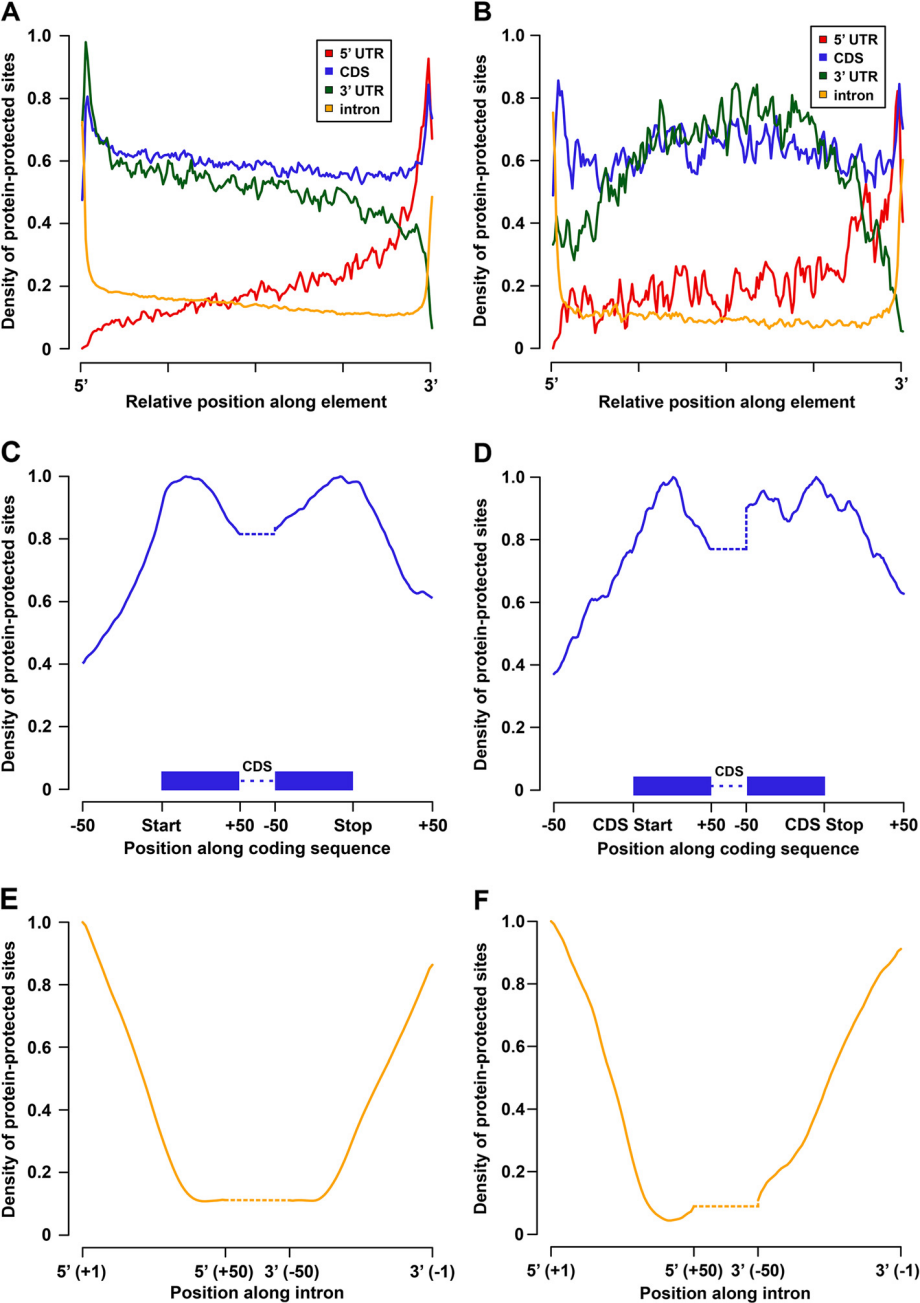
**The landscape of protein-binding site density. (A,B)** Average PPS density for formaldehyde **(A)** and UV **(B)** cross-linking experiments across 100 equally spaced bins in various genic regions. Values are normalized separately for each genic region (for example, intron). **(C,D)** Average PPS density for formaldehyde **(C)** and UV **(D)** cross-linking experiments within 50 nucleotides of CDS ends. **(E,F)** Average PPS density for formaldehyde **(E)** and UV **(F)** cross-linking experiments within the first and last 50 nucleotides of introns. Dotted lines in **(C,D,E,F)** represent the remaining (unanalyzed) length of each element. CDS, coding sequence; PPS, protein-protected site; UTR, untranslated region.

Given the importance of RBP binding within different regions of mRNAs, we decided to determine the density of protein-binding sites within specific regions of protein-coding transcripts (Figure 4 and Additional file 9). To do this, we first identified PPSs within each annotated CDS, 5′ UTR, 3′ UTR, and intronic region and calculated the relative distribution of binding sites across these regions (Figure 4A,B and Additional file 9A). We corrected for the average length of each region to obtain a global view of relative binding between regions. We also calculated PPS coverage on a per nucleotide basis for specific subregions of protein-coding mRNAs (Figure 4C,D,E,F, and Additional file 9B,C).

Applying this approach to PPSs identified with formaldehyde cross-linking, we observed similarly high levels of binding within the entirety of the CDS and 3′ UTR of protein-coding transcripts with an enrichment for binding events occurring at and near the start and stop codons (Figure 4A,C). This enrichment was particularly evident when interrogating the PPS density over the start and stop codons on a per nucleotide basis (Figure 4C). Similar enrichments leading to the start of the CDS were identified when defining PPS densities in the 5′ UTR. We also found that the overall protein-binding density was lower in the 5′ UTR compared to the CDS and 3′ UTR (Figure 4A). The observed enrichment of PPSs at the CDS start and stop codon regions likely reflects ribosome binding, as was previously observed by others [27,28].

Overall similar patterns of RBP binding were also observed for the UV-cross-linking and no-cross-linking experiments (Figure 4B and Additional file 9A). The two exceptions were that UV-cross-linked and non-cross-linked RBP-binding densities across the 3′ UTR peaked near the middle of this region (Figure 4B and Additional file 9A), and the interaction profile directly over the start codon displayed a minor depletion in protein binding in these experiments (Figure 4D and Additional file 9B). These results likely reflect the differential cross-linking specificities of formaldehyde and UV, and support the use of multiple cross-linkers in the comprehensive identification of RBP-binding sites.

Given the ability of PIP-seq to capture unprocessed RNAs, we also investigated RBP-binding density across introns. Unsurprisingly, we observed most binding events proximal to the 5′ and 3′ splice sites (Figure 4A,B and Additional file 9A). This was consistent across cross-linkers and is likely due to extensive association with the lariat formation machinery proximal to the splice sites. At single-base resolution, we located the beginning of this enrichment starting 40 nucleotides away from each splice site, consistent with the binding location of RNA splicing factors (Figure 4E,F and Additional file 9C). In total, our results indicate that PIP-seq gives a comprehensive view of RNA–protein interaction site densities in all portions of mature as well as unprocessed mRNAs, especially when multiple cross-linking agents are employed.

### PIP-seq uncovers known and novel RNA–protein interaction motifs and provides evidence for the post-transcriptional operon hypothesis

Given that PPSs correspond to protein-bound RNA sequences (Figure 2), we sought to gain insights into the sequence elements that are enriched within RNA–protein interaction sites in the HeLa transcriptome. To do this, we employed the MEME (Multiple EM for Motif Elicitation) algorithm [35] on PPSs partitioned by specific region (for example, 5′ UTR, 3′ UTR, CDS and intron). Because we could not rule out ribosome binding at start and stop codons, we additionally removed the first and last exons of each CDS. Using this approach, we identified previously known binding motifs including sequences similar to the LIN28 binding motif [24] and U-rich sequences (Additional file 10). We also identified numerous putative RBP-binding motifs, some of which are particularly interesting because they are long (approximately 20 nucleotides) and contain multiple strong consensus sequences flanked by weaker ones (3′ UTR motifs 4 and 31 and intron motifs 1 and 13) (Additional file 10). These motifs may correspond to binding by multiple RNA-binding domains (for example, RRM) of a single protein or by a complex of multiple RBPs. Importantly, motifs with this signature have not been previously reported in CLIP-seq and PAR-CLIP data. In addition, we identified at least one sequence that displayed a high degree of self-complementarity (3′ UTR motif 1). This is surprising, given that MEME does not use RNA secondary structure as a search feature when identifying motifs from a set of given sequences. These findings underscore the utility of PIP-seq and its use of multiple structure-specific nucleases to uncover hidden features of the protein-interacting transcriptome.

**Figure 5:**
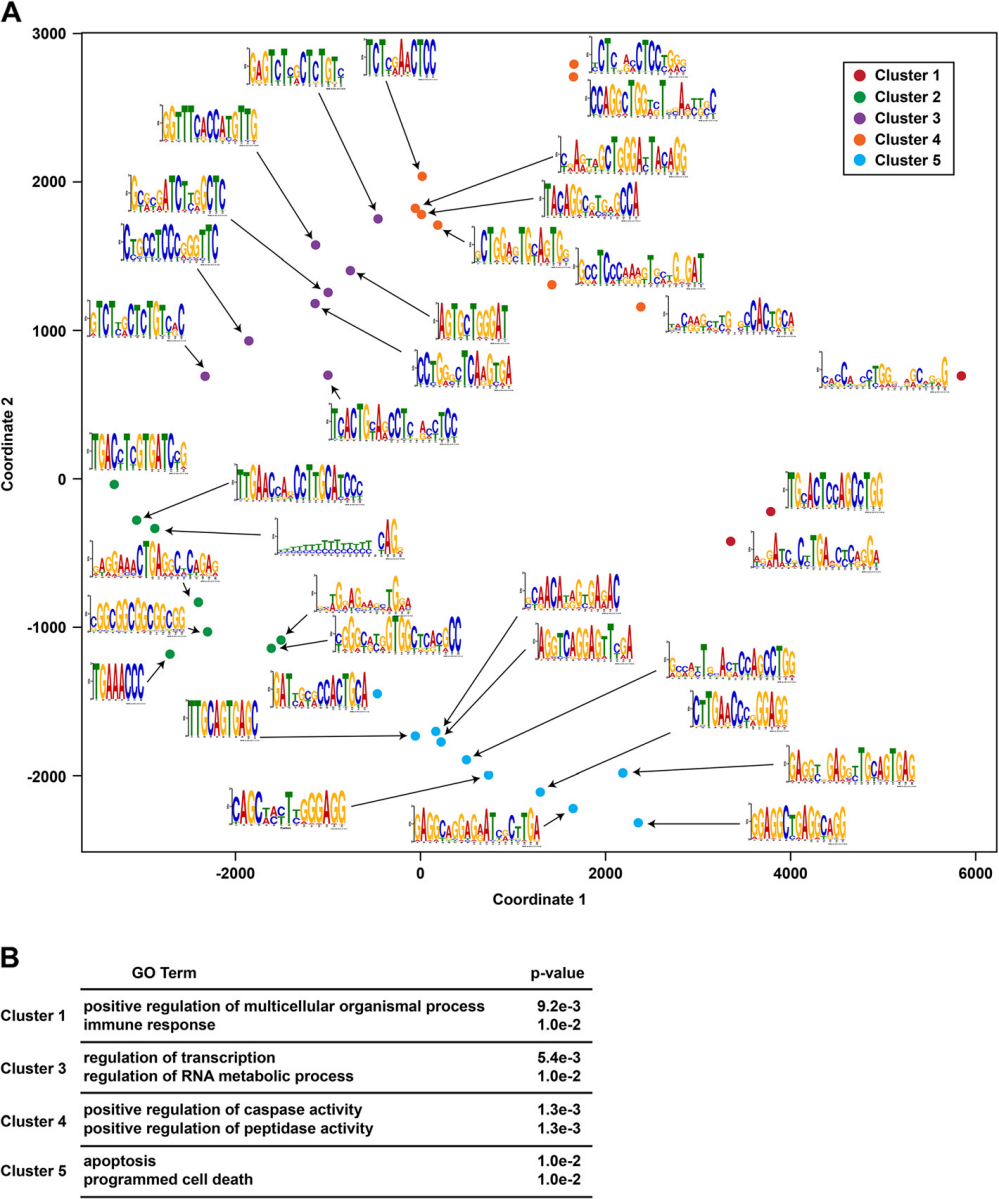
**PIP-seq uncovers protein-bound sequence motifs that co-occur in groups of functionally related transcripts. (A)** MDS analysis of RBP-bound motif co-occurrence in human mRNAs. The motifs used for this study were identified by a MEME-based analysis of PPS sequences. Sequences for all of the motifs used in this analysis can be found in Additional file 10. Colors indicate cluster membership as defined by *k*-means clustering (*k* = 5). **(B)** The most significantly enriched biological processes (and corresponding *P* value) for target transcripts, where the specified clusters of motifs identified in **(A)** are co-bound. MDS, multidimensional scaling; PIP-seq, protein interaction profile sequencing; PPS, protein-protected site; RBP, RNA-binding protein.

Although RNAs are thought to be bound and regulated by multiple RBPs, very little is known about these interactions and the relations between specific RBPs and their corresponding sequence motifs. To address this, we interrogated the interactions between putative RBP-binding motifs (Figure 5A) discovered by our PIP-seq approach, since these are protein-bound sequences in HeLa cells. To do this, we first identified all instances of each motif within the global set of identified PPSs on target RNAs using FIMO [36]. We collapsed motifs with similar sequences and excluded those that were long (approximately 20 nucleotides) and non-degenerate because these likely represent repetitive sequences instead of true binding motifs. We then quantified the co-binding of the remaining motifs (approximately 40) within all protein-coding mRNAs by counting the number of transcripts on which each pair of motifs was jointly found within PPSs. We then used *k*-means clustering of the resultant weighted adjacency matrix and identified five clusters of motifs that interact on highly similar sets of target mRNAs (Figure 5A). These findings indicate that many mRNAs contain numerous RBP-interacting motifs within their sequences and that coordinated binding of RBPs to specific target transcripts may represent a general phenomenon of cellular RNA–protein interactions, as was previously proposed by the post-transcriptional operon hypothesis [9,10].

We also used DAVID [37] to interrogate over-represented biological processes for RNAs that contained binding events for each motif from the five clusters identified in the *k*-means analysis (Figure 5A, Clusters 1, 3 to 5). It is of note that the motifs in Cluster 2 did not co-occur in a large enough group of bound transcripts to allow meaningful gene ontology (GO) analysis. We found that the most highly over-represented functional terms for the RNAs that contained these co-occurring sequence motifs in HeLa Clusters 1, 3 to 5 were related to distinct processes, including developmental processes and immunity (Cluster 1), caspase activity and apoptosis (Clusters 4 and 5, respectively), as well as regulation of transcription and RNA metabolic processes (Cluster 3) (Figure 5B). These results suggest that there are distinct groups of RBP recognition motifs that are involved in the post-transcriptional regulation of various collections of mRNAs encoding functionally related proteins.

### Disease-linked SNPs correlate with protein-bound RNA sequences

**Figure 6:**
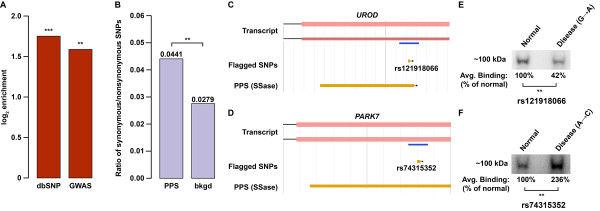
**Disease-associated SNPs are enriched within PPSs in human transcripts. (A)** Enrichment of disease-associated SNPs from dbSNP build 137 and the NHGRI GWAS Catalog in PPSs versus background. *** denotes *P* → 0 and ** denotes *P* < 0.001 (chi-squared test). **(B)** Ratio of synonymous to non-synonymous SNPs in PPSs versus background. ** denotes *P* < 0.001 (chi-squared test). **(C,D)** Two examples of disease-related SNPs found in *UROD***(C)** and *PARK7***(D)** that overlap with PPSs identified by PIP-seq in HeLa cells using ssRNase treatment (SSase). The screenshots are from our PIP-seq browser [46]. The *UROD* and *PARK7* SNPs (as indicated in the flagged SNPs track) are used in the analyses shown in **(E)** and **(F)**, respectively. A blue line below the transcript model denotes the regions used for the analyses in **(E)** and **(F)**. **(E,F)** UV-cross-linking analysis of normal compared to disease-related SNPs using probes with only the specific base pair substitution, as specified in parentheses next to the disease label, and protein lysates from HeLa cells. The rs121918066 **(E)** and rs74315352 **(F)** SNPs associated with porphyria cutanea tarda and early-onset Parkinson’s disease, respectively, were used in this analysis. Representative images for three replicate experiments. ** denotes *P* < 0.001 (one-tailed *t*-test). bkgd, background; PIP-seq, protein interaction profile sequencing; PPS, protein-protected site; SNP, single nucleotide polymorphism; SSase, ssRNase treatment.

A growing set of evidence suggests that multiple RNA-level mechanisms, some of which depend upon RNA–protein interactions, are the means by which particular single nucleotide polymorphisms (SNPs) in mRNAs effect human disease phenotypes [38-41]. In support of this, we found PPSs to be enriched in disease-associated SNPs from dbSNP build 137 and the NHGRI GWAS Catalog (Figure 6A). Furthermore, the ratio of synonymous to non-synonymous SNPs was also significantly higher within PPSs compared with the expressed transcriptome background (Figure 6B, *P* = 9.8 × 10^–4^), lending further support to the notion that disruption of RNA–protein interactions underlies the disease mechanism of the polymorphisms in question.

To verify that disease-related human SNPs can affect RBP–RNA interactions, we used UV-cross-linking analyses with 38-nucleotide-long RNA probes containing either the normal or disease-associated variant at their center. For these analyses, we focused on two specific SNPs that are associated with porphyria cutanea tarda and early-onset Parkinson’s disease (rs121918066 and rs74315352, respectively) (Additional file 11). We found that both disease-associated SNPs tested had significant effects on specific RBP–RNA interactions (*P* < 0.001) (Figure 6C,D). In fact, we found that rs121918066 disrupted, while rs74315352 enhanced, specific interactions with an RBP complex. These findings revealed that disease-associated SNPs that reside within RBP-binding sites can affect the interaction between proteins and their target RNAs. In total, these results suggest that modulation of RBP interactions may be a significant RNA-level disease mechanism in humans.

## Conclusions

In general, the global architecture of RNA–protein interactions within the population of both unprocessed and mature RNA molecules is still poorly characterized [1,11,12]. We described a novel RNase-mediated protein footprint sequencing approach (PIP-seq), which globally identifies RNA–protein interactions for numerous RBPs in the human transcriptome with a single experiment (Figure 1A). Our approach is similar to other recently published methodologies [27], but in addition to polyA-containing mature mRNAs we also provide a view of RNA–protein interaction sites in unprocessed mRNAs (that is, introns). Additionally, our approach is widely applicable to all samples and organisms since it is not dependent on the incorporation of non-natural nucleotides or UV cross-linking.

Analysis of the PPSs uncovered by our approach allowed us to identify significant levels of known and novel RNA–protein interaction sites and sequence motifs. By comparing across cross-linkers and RNases, we demonstrated that each uncovers specific subsets of protein-bound sequences. This supports the use of multiple reagents for obtaining a comprehensive analysis of the protein-bound transcriptome in eukaryotic organisms.

Using the RNA sequences identified as being protein bound in the HeLa cell transcriptome by PIP-seq, we uncovered a large set of putative RBP-binding motifs. Based on their size and sequence characteristics, it is likely that many of these motifs correspond to binding sites for RBPs that interact with target RNAs through multiple RNA-binding domains or complexes of multiple RBPs. We used these identified RBP-bound motifs to investigate the interaction between RBPs within target mRNAs and offer insights into mRNP organization in the human transcriptome. This study is one of the first to examine comprehensively the co-binding by RBPs with specific target mRNAs. Our findings are an important resource for investigating the binding of groups of RBPs to collections of mRNAs encoding proteins functioning in specific biological processes. These sequences can be used to identify the interacting proteins so that their effects on post-transcriptional regulation can be further studied.

Finally, we observed a significant overlap of PPSs with disease-linked SNPs obtained from two different sources (dbSNP build 137 and NHGRI GWAS Catalog [42]), and validated these results using UV-cross-linking experiments that demonstrated disease-linked SNPs could disrupt or enhance RBP–RNA interactions. Thus, determining the molecular details behind each disease-associated SNP that affects an RNA–RBP interaction will be an important future research endeavor. It is also worth noting that our findings point to the intriguing possibility that PIP-seq could be used in conjunction with genome-wide association studies to screen for synonymous mutations that may be causal via altering of any number of RNA–protein interactions in affected tissues. Such a tool would be extremely valuable in mechanistic, pharmacogenomic and therapeutic studies of disease-associated polymorphisms. In summary, we present a powerful method that will be important for future studies of RNA–protein interaction site dynamics in multiple eukaryotic organisms and in important biological contexts.

## Materials and methods

### Cell lines

For these experiments, HeLa cells were seeded in 15-cm standard Corning tissue-culture treated culture dishes (Sigma, St Louis, MO), grown to 90% confluence (approximately 18 million cells) in DMEM media (Life Technologies, San Diego, CA) supplemented with L-glutamine, 4.5 g/L D-glucose, 10% fetal bovine serum (FBS (Atlanta Biologics, Atlanta, GA)) and Pen/Strep (Fisher Scientific, Waltham, MA).

### Cross-linking experiments

For formaldehyde cross-linking, a 37% formaldehyde solution (Sigma, St. Louis, MO) was added drop-wise with mixing directly to cell culture dishes containing 90% confluent cells to a final concentration of 1% and incubated at room temperature for 10 minutes. Next, 1 M glycine (Sigma, St Louis, MO) was added to a final concentration of 125 mM and incubated for an additional 5 minutes with mixing. Then, cells were washed twice with ice-cold PBS and collected. Finally, cells were pelleted and frozen until the PIP-seq digestions were performed. For UV-cross-linking experiments, 90% confluent cells were washed twice with ice-cold PBS and resuspended in 5 mL of PBS. Cell culture dishes were placed in a UV Stratalinker 2400 (Agilent Technologies, New Castle, DE) with the lid removed and irradiated with UV-C (254 nm) once at 400 mJ/cm^2^. The cross-linked cells were collected by scraping, pelleted and then frozen until used.

### PIP-seq library preparation

To begin, we lysed the cell pellets in RIP buffer (25 mM Tris–HCl, pH = 7.4; 150 mM KCl, 5 mM EDTA, pH = 7.5; 0.5% NP40; 10 μM DTT; 1 tablet protease inhibitors/10 mL) and ground them manually (850 μl of RIP was used per 10 million cells). The resulting cell lysate was treated with RNase-free DNase (Qiagen, Valencia, CA). Subsequently, these DNA-depleted lysates were split and treated with either 100 U/mL of a single-stranded RNase (ssRNase) (RNaseONE (Promega, Madison, WI)) with 200 μg/mL BSA in 1× RNaseONE buffer for 1 hour at room temperature, or 2.5 U/mL of a double-stranded RNase (dsRNase) (RNaseV1 (Ambion, Austin, TX)) in 1× RNA structure buffer for 1 hour at 37°C as previously described [29,30] (see Figure 1A for a schematic description). The proteins were then denatured and digested by treatment with 1% SDS and 0.1 mg/mL proteinase K (Roche, Basel, Switzerland) for 15 minutes at room temperature. We used two cell lysates for these experiments: one treated with the ssRNase and the other with dsRNase. For the formaldehyde-cross-linking experiments, proteinase digestion was followed by a 2-hour incubation at 65°C to reverse the cross-links, whereas for the UV-cross-linking experiments, RNA was liberated from protein by retreating the lysates with 1% SDS and 1 mg/mL proteinase K for 30 minutes.

To determine whether nuclease-resistant regions in RNAs are due to protein binding or specific secondary structures, we also determined the digestion patterns of ds- and ssRNases in the absence of bound proteins. To do this, we performed the identical treatments as described above except that the cross-linked cellular lysates were treated with 1% SDS and 0.1 mg/mL proteinase K (Roche, Basel, Switzerland) and ethanol-precipitated prior to being treated with the two RNases. In this way, the SDS and proteinase K solubilized and digested the proteins allowing us to deduce PPSs within all detectable RNAs in the cells of interest (see Figure 1A for a schematic).

The digested RNA was then isolated using the Qiagen miRNeasy RNA isolation kit following the manufacturer’s protocol (Qiagen, Valencia, CA). Finally, the purified RNA was used as the substrate for strand-specific sequencing library preparation, as previously described [29,30], with the exception that we also included DSN library normalization per the manufacturer’s instructions (Illumina, San Diego, CA). Briefly, 100 ng of the final library was denatured at 95°C and then annealed for 5 hours at 68°C. Next, 2 μl of DSN enzyme (1 U/μl) was used to deplete the re-annealed duplexes. All of the RNase footprint libraries (a total of four for each replicate: ss- and dsRNase treatments, footprint and RNase digestion controls) were sequenced on an Illumina HiSeq2000 using the standard protocols for 50-bp single-read sequencing.

### Read processing and alignment

PIP-seq reads were first trimmed to remove 3′ sequencing adapters using cutadapt (version 1.0 with parameters -e 0.06 –O 6 -m 14). The resulting trimmed sequences were collapsed to unique reads and aligned to the human genome (hg19) using Tophat (version 2.0.9 with parameters --read-mismatches 2 --read-edit-dist 2 --max-multihits 10 --b2-very-sensitive --transcriptome-max-hits 10 --no-coverage-search --no-novel-juncs). PCR duplicates were collapsed to single reads for all subsequent analyses.

### Identification of PPSs

PPSs were identified using a modified version of the CSAR software package [34]. Specifically, read coverage values were calculated for each base position in the genome and a Poisson test was used to compute an enrichment score for footprint versus RNase digestion control libraries. PPSs were then called as described [34] with an FDR of 5%.

### PPS saturation analysis

Mapped reads from chromosome 9 of formaldehyde-cross-linked ssRNase-treated PIP-seq replicate 1 libraries were randomly subsampled at 10% to 90% by a custom Perl script. CSAR was used to identify PPSs as described and the total number of PPSs was plotted as a function of subsample size.

### Validation by comparison with CLIP-seq, PAR-CLIP and gPAR-CLIP data

iCLIP, PAR-CLIP, and CLIP-seq datasets were compiled from sources as referenced and overlapped with PPSs. The significance of overlaps with PPSs was assessed using a chi-squared test compared to an expressed transcriptome background. To compute a background distribution for the number of T > C transversions, we generated ten random sets of genomic intervals with the same size distribution as PPSs. These random intervals were selected from a background of actively transcribed regions (defined using bgrSegmenter [43] with parameters: threshold = 10, maxGap = 10 and minRun = 15).

### Functional analysis of PPSs

Gene annotations were downloaded from the UCSC Genome Browser (RefSeq Genes, wgRna, rnaGene, lncRNA), and miRBase release 18 was used for the microRNA annotations. PPS annotation was done ‘greedily’, such that all functional annotations that overlapped with a given PPS were counted equally. Conservation was assessed by computing average SiPhy-π log-odds [44] scores within PPSs and in equal-sized regions immediately upstream and downstream of each PPS.

### Motif and co-occurrence analysis

MEME [35] was used to identify enriched RBP interaction motifs with parameters –dna –nmotifs 100 –evt 0.01 –maxsize 100000000. Motif co-occurrence was defined at the transcript level, and *k*-means clustering of the resultant weighted adjacency matrix was used to identify modules of co-occurring motifs. We set *k* = 5 based on manual inspection of clusters on a multidimensional scaling (MDS) plot of the adjacency matrix. GO analysis was performed using DAVID [37].

### Analysis of SNPs and disease associations

Clinically associated SNPs (snp137Flagged) were downloaded from the UCSC Table Browser. We also downloaded the NHGRI GWAS Catalog [42] of disease-linked SNPs. Background distributions refer to the incidence of each dataset within the same genic regions as those of the PPSs in each analysis. Significance was assessed using a chi-squared test.

### UV-cross-linking analysis of disease-associated SNPs

We generated asymmetric oligonucleotide hybrids for *in vitro* transcription by annealing T7 sense DNA oligonucleotides (TAATACGACTCACTATAGGG) to antisense probe sequences fused to the antisense T7 (aT7) sequence (rs74315352 normal: CTTGTAAGAATCAGGCCGtCTTTTTCCACACGATTCTC(aT7), rs74315352 disease: CTTGTAAGAATCAGGCCGgCTTTTTCCACACGATTCTC(aT7), rs121918066 normal: CCCAGGTTGGCAATGTAGcGATGTGGTCCAAAGTCATC(aT7), rs121918066 disease: CCCAGGTTGGCAATGTAGtGATGTGGTCCAAAGTCATC(aT7)) (IDT, San Jose, CA). Each hybrid reaction was incubated at 95°C for 5 minutes and cooled to 25°C by step-wise increments of 1°C/minute.

*In vitro* transcription reactions were performed by adding 1 μg of the asymmetric oligonucleotide hybrids (see above) to a 25 μL transcription reaction comprising 1× T7 RNA Transcription buffer (NEB, Cambridge, MA), 36 μM uridine triphosphate (UTP) (for rs74315352) or 36 μM cytidine triphosphate (CTP) (for rs121918066), 264 μM each of ATP, CTP and guanosine triphosphate (GTP) (for rs74315352) or 264 μM each of ATP, UTP and GTP (for rs121918066), 0.04 mCi ^32^P UTP (for rs74315352) or 0.04 mCi ^32^P CTP (for rs121918066), 10 nM DTT, 40 U RNaseOUT (Invitroge, Carlsbad, CA), and 75 U of T7 RNA polymerase. The reactions were incubated at 37°C for 2 hours. DNA was digested with four units of Turbo DNase (Invitrogen, Carlsbad, CA) at 37°C for 20 minutes. RNA probes were chloroform-extracted and precipitated. The amount of a labeled RNA probe was determined by 15% TBE-urea gel electrophoresis followed by phosphor-imaging and densitometry. Normal and disease RNA probes were normalized to equal activities and used for subsequent analysis.

Equal concentrations of each RNA probe (approximately 10% of the total from *in vitro* transcription) were added to separate 10.2 μL binding reactions comprising 0.2 mM Tris pH 7.5, 0.02 mM EDTA, 40 mM KCl, 1.3% polyvinyl alcohol, 25 ng/μl tRNA, 3 mM MgCl_2_, 1 mM ATP, 50 mM creatine phosphate and 1.5 μg/μl HeLa whole cell lysate in RIP buffer (25 mM Tris–HCl, pH = 7.4; 150 mM KCl, 5 mM EDTA, pH = 7.5; 0.5% NP40; 10 μM DTT; 1 tablet protease inhibitors/10 mL) and incubated at 30°C for 20 minutes. The binding reaction was then subjected to UV cross-linking for 20 minutes using a 254-nm UV lamp (Mineralight Lamp Model R-52G (UVP, Upland, CA)). To digest unbound RNA, each reaction was incubated with 20 U RNase T1 and 8 μg RNase A at 37°C for 20 minutes. RNA-bound proteins were denatured in 1× SDS sample buffer and 1 mM β-mercaptoethanol and boiled for 5 minutes. Samples were separated on NuPAGE 3% to 8% Tris-acetate gel (Invitrogen, Carlsbad, CA) at 130 V for 1.5 hrs. Phosphor-imaging and densitometry were used to visualize and quantify protein-bound RNA, respectively.

### Accession numbers

All PIP-seq data from our analyses were deposited in GEO under the accession GSE49309. All of our data (files of all identified PPSs, complete lists of overrepresented motifs, GO analyses and so on) can also be accessed at [45]. The web browsers used for visualization of all PPSs and our analyzed and raw sequencing data can be found at [46] for jbrowse and at [47] for the UCSC genome browser.

## Abbreviations

bp: Base pair; BSA: Bovine serum albumin; CDS: Coding sequence; CLIP-seq: Cross-linking and immunoprecipitation sequencing; CTP: Cytidine triphosphate; DSN: Duplex-specific nuclease; dsRNA: Double-stranded RNA; dsRNase: Double-stranded RNase; FDR: False discovery rate; GO: Gene ontology; gPAR-CLIP: Global photoactivatable ribonucleoside cross-linking and immunoprecipitation; GTP: Guanosine triphosphate; lncRNA: Long non-coding RNA; MDS: Multidimensional scaling; mRNA: Messenger RNA; ncRNA: Non-coding RNA; NS: Not significant; PAR-CLIP: Photoactivatable ribonucleoside cross-linking and immunoprecipitation; PBS: Phosphate-buffered saline; PCR: Polymerase chain reaction; PIP-seq: Protein interaction profile sequencing; polyA: Poly-adenylated; PPS: Protein-protected site; RBD: RNA-binding domain; RBP: RNA-binding protein; RNase: Ribonuclease; RNP: Ribonucleoprotein complex; SNP: Single nucleotide polymorphism; ssRNA: Single-stranded RNA; ssRNase: Single-stranded RNase; UTP: Uridine triphosphate; UTR: Untranslated region.

## Competing interests

The authors declare that they have no competing interests.

## Authors’ contributions

IS, FL, LG, CT, JLR and BDG conceived the study and designed the experiments. IS, FL, AA and BDG performed the experiments. IS, FL and BDG analyzed the data and wrote the paper with assistance from the other authors. All authors have read and approved the manuscript for publication.

## Supplementary Material

Additional file 1: **PIP-seq library characteristics.** Information on PIP-seq libraries, including sequencing reads, processing, mapping and PPS identification. Click here for file

Additional file 2 **PIP-seq reveals protein-binding sites throughout the human transcriptome (related to Figure** 1**).** (A,B,C,D,E) Screenshots show PIP-seq reads for the formaldehyde-cross-linked ssRNase-treated footprint (top sequencing track) and RNase digestion control libraries (bottom sequencing track). Blocks indicate regions identified as PPSs in each of the three replicates. Chromosomal coordinates, UCSC gene tracks (including alternative events), and PhyloP conservation scores are included (as labeled). The screenshots are from our PIP-seq browser [47]. Examples include four protein-coding genes (A to D) and an lncRNA gene (E). (F) Number of PPSs identified in subsets of total reads from human chromosome 9 for the formaldehyde-cross-linked ssRNase-treated libraries. Click here for file

Additional file 3 **Summary statistics for PPSs identified by UV-cross-linked and non-cross-linked experiments (related to Figure** 1**).** (A) Absolute distribution of PPSs throughout RNA species identified using no cross-linking. (B) Average PPS count per RNA molecule (classified by type (mRNA and lncRNA) and transcript region (for example, 5′ UTR)) identified using no cross-linking. Percentages indicate the fraction of each RNA type or region that contains PPS information. (C) Average expression (*y*-axis) of human mRNAs separated by total number of PPSs identified in their sequence (*x*-axis) for UV-cross-linked experiments. (D) As (C), but for PPSs identified using no cross-linking. Click here for file

Additional file 4 **Correlation of PIP-seq read counts (related to Figure** 2**).** Correlation in read counts between additional formaldehyde- (A and B) and UV-cross-linked (C) PIP-seq replicates as labeled. Click here for file

Additional file 5 **PIP-seq is a reproducible approach (related to Figure** 2**).** (A) Overlap in PPS calls between additional formaldehyde-cross-linked ssRNase-treated (blue) and dsRNase-treated (green) PIP-seq replicates. (B) Overlap in PPS calls between two replicates of UV-cross-linked ssRNase-treated (blue) and dsRNase-treated (green) PIP-seq replicates. (C) Overlap in PPS calls between formaldehyde-cross-linked ssRNase-treated and dsRNase-treated PIP-seq samples. (D – E) As (C), but for UV-cross-linked replicates (D) and the non-cross-linked experiment (E). Click here for file

Additional file 6 **Validation of PIP-seq by comparison with previously published RBP binding site datasets.** (A) Overlap between PPSs identified from two UV-cross-linked PIP-seq samples and various CLIP datasets. Values are shown as log_2_ enrichment over shuffled background distributions. (B) As (A), but for a non-cross-linked PIP-seq sample. *** denotes *P* < 2.2 × 10^-16^ (chi-squared test). (C) Overlap between UV-cross-linked PPSs and 40-nucleotide T > C transversion event-containing loci from the gPAR-CLIP dataset (T > C transversion events less than 40 bp apart were merged to generate a dataset comparable to PPSs). (D) As (C), but for PPSs identified with no cross-linking. (E) Number of T > C transversion events per PPS identified using UV cross-linking (magenta) versus shuffled regions (gray). Values for the number of events per shuffled region are the average from ten random shuffles. (F) As (E), but for PPSs identified with no cross-linking. Click here for file

Additional file 7 **Characterization of PPSs identified by UV-cross-linking and no-cross-linking PIP-seq experiments (related to Figure** 3**).** (A) Distribution of ssRNase-treated (blue) and dsRNase-treated (green) PPS sizes from UV-cross-linked samples. Dashed lines represent mean PPS sizes (ssRNase, blue line and dsRNase, green line). (B) As (A), but for non-cross-linked PPSs. (C) Genomic distribution of UV-cross-linked PPS density, measured as PPS base coverage normalized to RNase digestion control read counts per genomic region. Proximal intron refers to 500 nucleotides at the 5′ and 3′ ends of introns. (D) As (C), but for non-cross-linked PPSs. (E) Fraction of base pairs covered by PPSs in 100 most highly expressed lncRNAs (orange bars) and expression-matched control mRNA 3′ UTRs (purple bars) for PIP-seq libraries made with ssRNase (ss) or dsRNase (ds) under the three different cross-linking conditions (as specified). Click here for file

Additional file 8 **PPSs identified by UV-cross-linking and no-cross-linking PIP-seq experiments are evolutionarily conserved (related to Figure** 3**).** (A,B) Cumulative distribution of average SiPhy-π scores in PPSs identified by UV cross-linking (A) and no cross-linking (B). PPSs (red) are compared to flanking sequences (gray). (C,D) Comparison of average SiPhy-π scores between PPSs identified by UV cross-linking (C) and no cross-linking (D). PPSs (red) are compared to flanking sequences (gray) for various genomic regions. (E,F) Average SiPhy-π score profiles across the first and last 25 nucleotides of PPSs identified by UV cross-linking (E) and no cross-linking (F), as well as 50 nucleotides upstream and downstream of exonic (green line), intronic (blue line) and lncRNA (orange line) PPSs. *** denotes *P* < 2.2 × 10^–16^ (chi-squared test). NS, not significant. Click here for file

Additional file 9 **Genomic distribution of PPSs identified by UV-cross-linking and no-cross-linking PIP-seq experiments (related to Figure** 4**).** (A) Average PPS density for no-cross-linking PIP-seq across 100 equally spaced bins in various genic regions. Values are normalized separately for each genic region (for example, intron). (B) Average PPS density for no-cross-linking PIP-seq within 50 nucleotides of CDS ends. (C) Average PPS density for no-cross-linking PIP-seq within the first and last 50 nucleotides of introns. Dotted lines in (B,C) represent the remaining (unanalyzed) length of each element. Click here for file

Additional file 10 **Motif and co-occurrence analyses. Motifs identified by MEME analyses of PPS sequences from specific subregions of protein-coding mRNAs.** Those motifs used in the co-occurrence analyses (Figure 5) are listed in this table. Click here for file

Additional file 11 **Table of disease-associated SNPs that overlap with PPSs.** Information on all flagged and GWAS SNPs overlapping PPSs identified using formaldehyde-cross-linked PIP-seq. Click here for file
