# Supplementary figures and images for: RNase-mediated protein footprint sequencing reveals protein-binding sites throughout the human transcriptome
Source: Genome Biol. 2014 Jan 7;15(1):R3. doi: 10.1186/gb-2014-15-1-r3 (PMC4053792; doi:10.1186/gb-2014-15-1-r3)

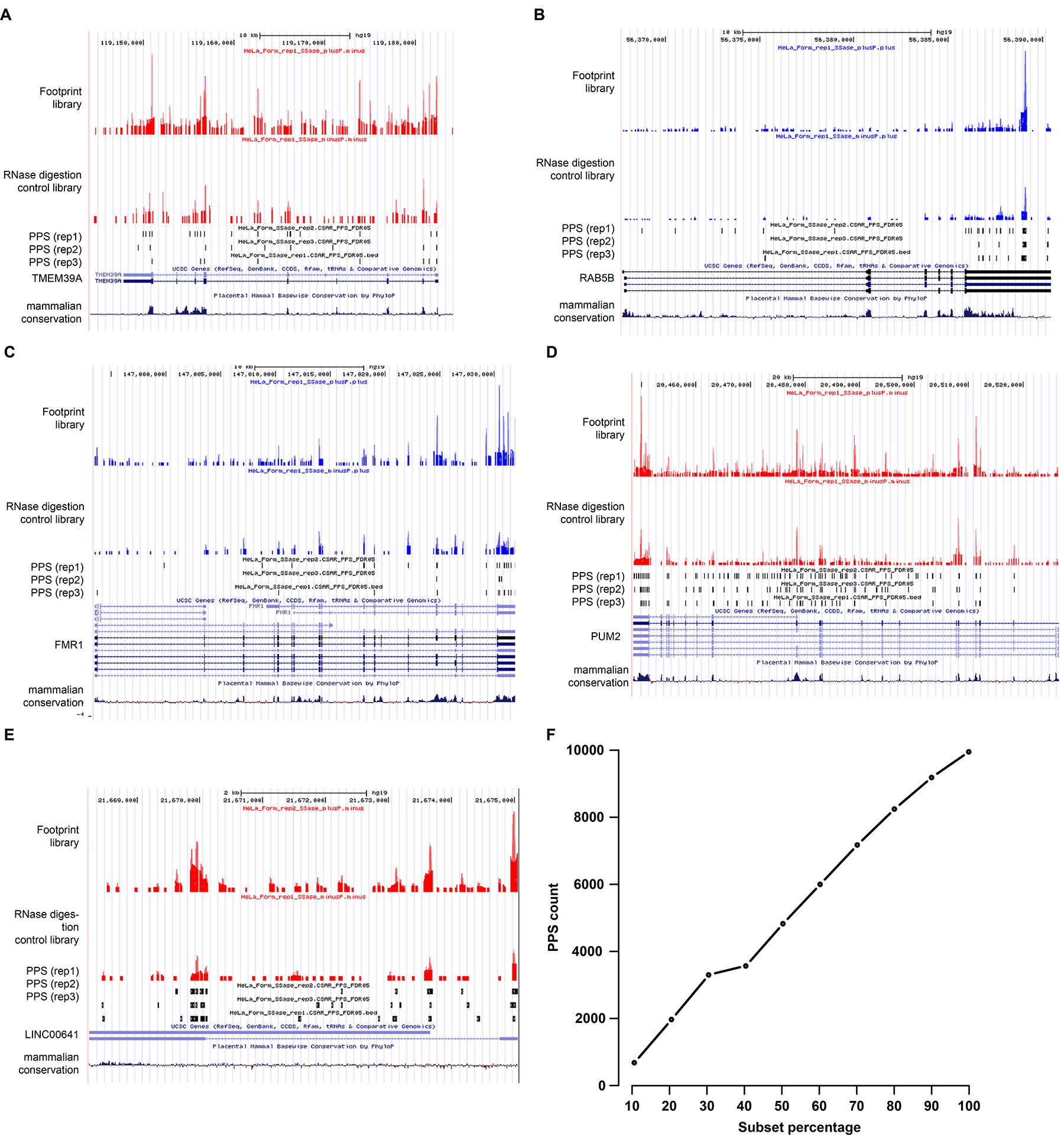

Supplement: Additional file 2 — PIP-seq reveals protein-binding sites throughout the human transcriptome (related to Figure 1). (A,B,C,D,E) Screenshots show PIP-seq reads for the formaldehyde-cross-linked ssRNase-treated footprint (top sequencing track) and RNase digestion control libraries (bottom sequencing track). Blocks indicate regions identified as PPSs in each of the three replicates. Chromosomal coordinates, UCSC gene tracks (including alternative events), and PhyloP conservation scores are included (as labeled). The screenshots are from our PIP-seq browser [47]. Examples include four protein-coding genes (A to D) and an lncRNA gene (E). (F) Number of PPSs identified in subsets of total reads from human chromosome 9 for the formaldehyde-cross-linked ssRNase-treated libraries. [file gb-2014-15-1-r3-S2.jpeg]

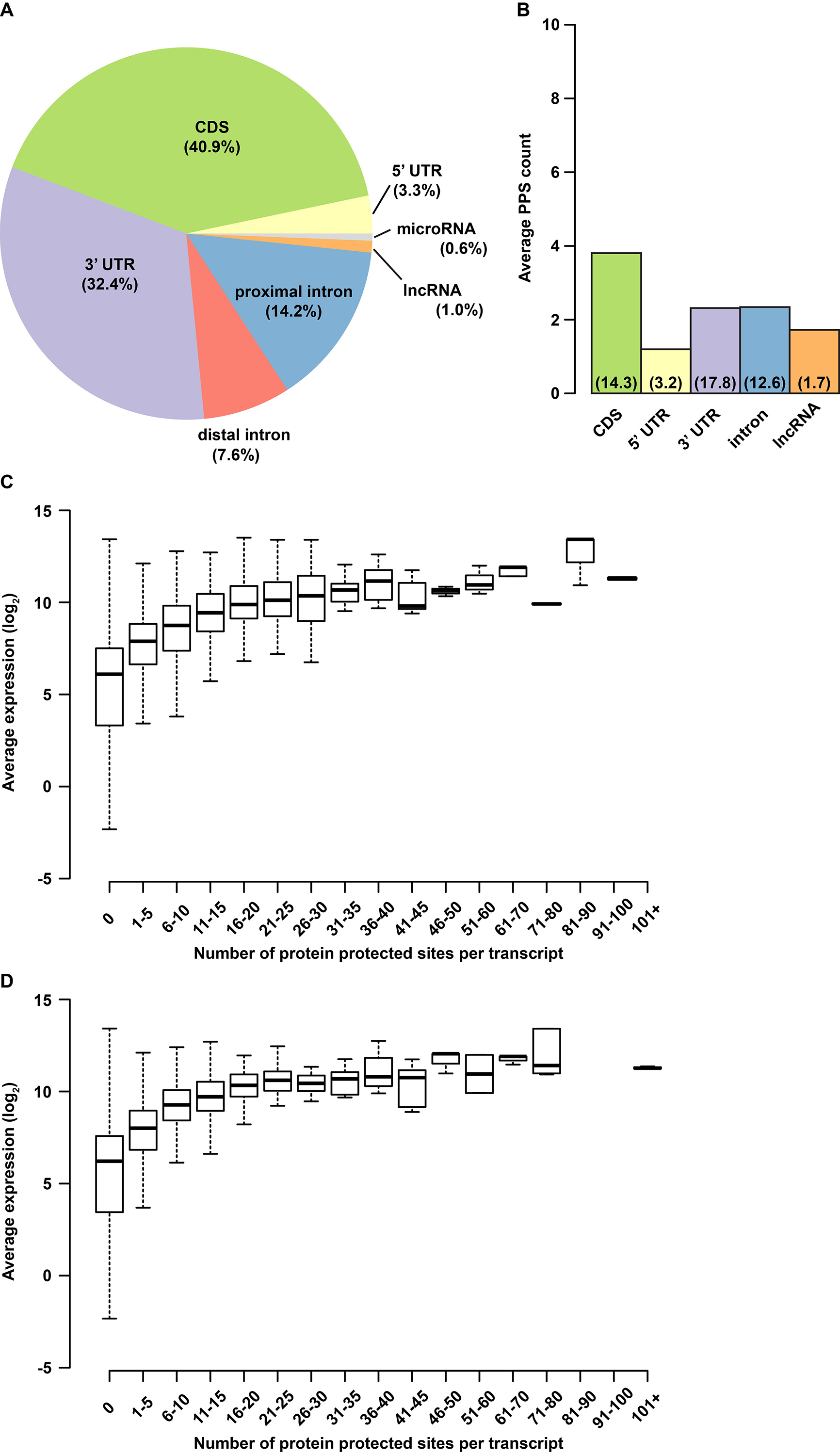

Supplement: Additional file 3 — Summary statistics for PPSs identified by UV-cross-linked and non-cross-linked experiments (related to Figure 1). (A) Absolute distribution of PPSs throughout RNA species identified using no cross-linking. (B) Average PPS count per RNA molecule (classified by type (mRNA and lncRNA) and transcript region (for example, 5′ UTR)) identified using no cross-linking. Percentages indicate the fraction of each RNA type or region that contains PPS information. (C) Average expression (y-axis) of human mRNAs separated by total number of PPSs identified in their sequence (x-axis) for UV-cross-linked experiments. (D) As (C), but for PPSs identified using no cross-linking. [file gb-2014-15-1-r3-S3.jpeg]

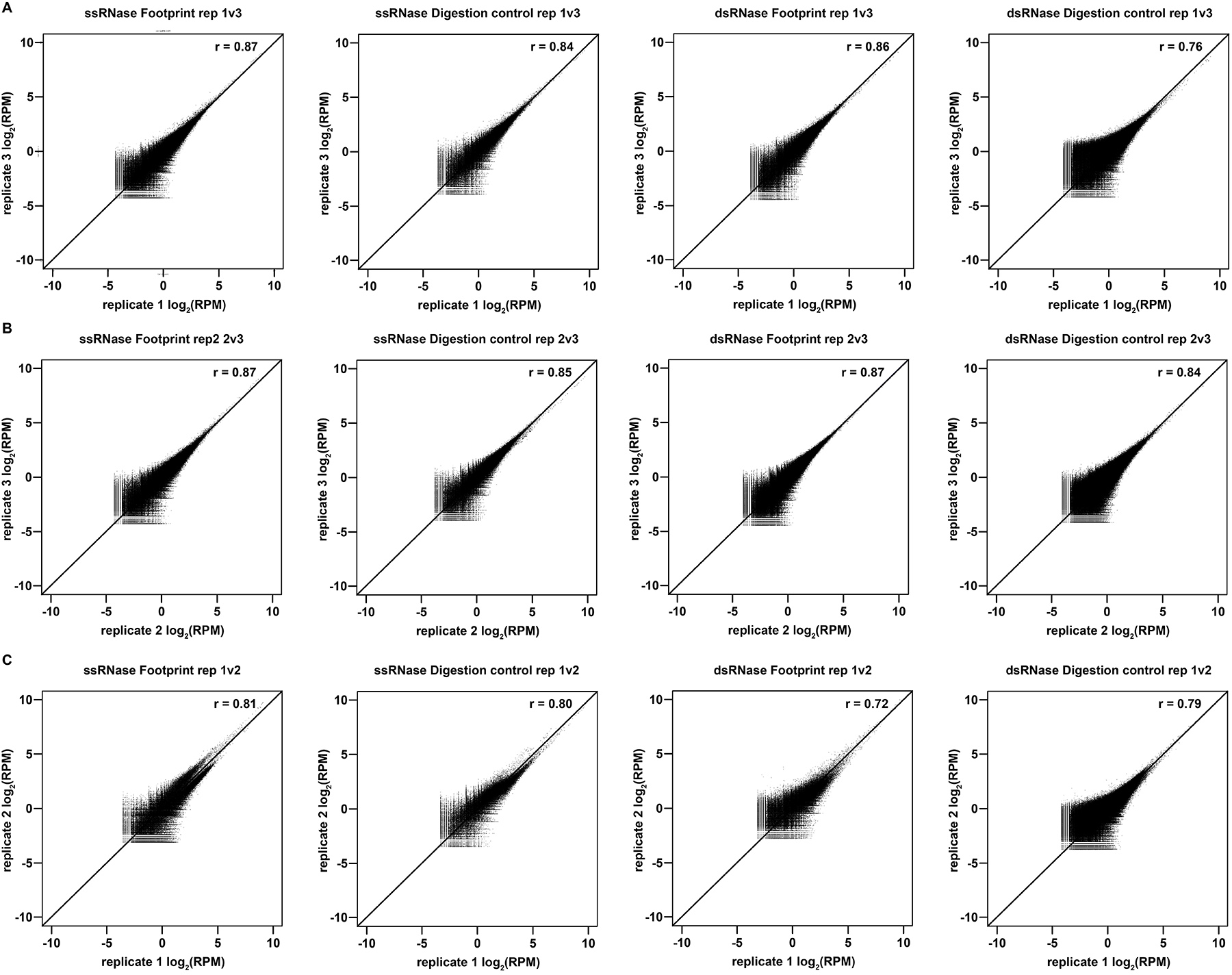

Supplement: Additional file 4 — Correlation of PIP-seq read counts (related to Figure 2). Correlation in read counts between additional formaldehyde- (A and B) and UV-cross-linked (C) PIP-seq replicates as labeled. [file gb-2014-15-1-r3-S4.jpeg]

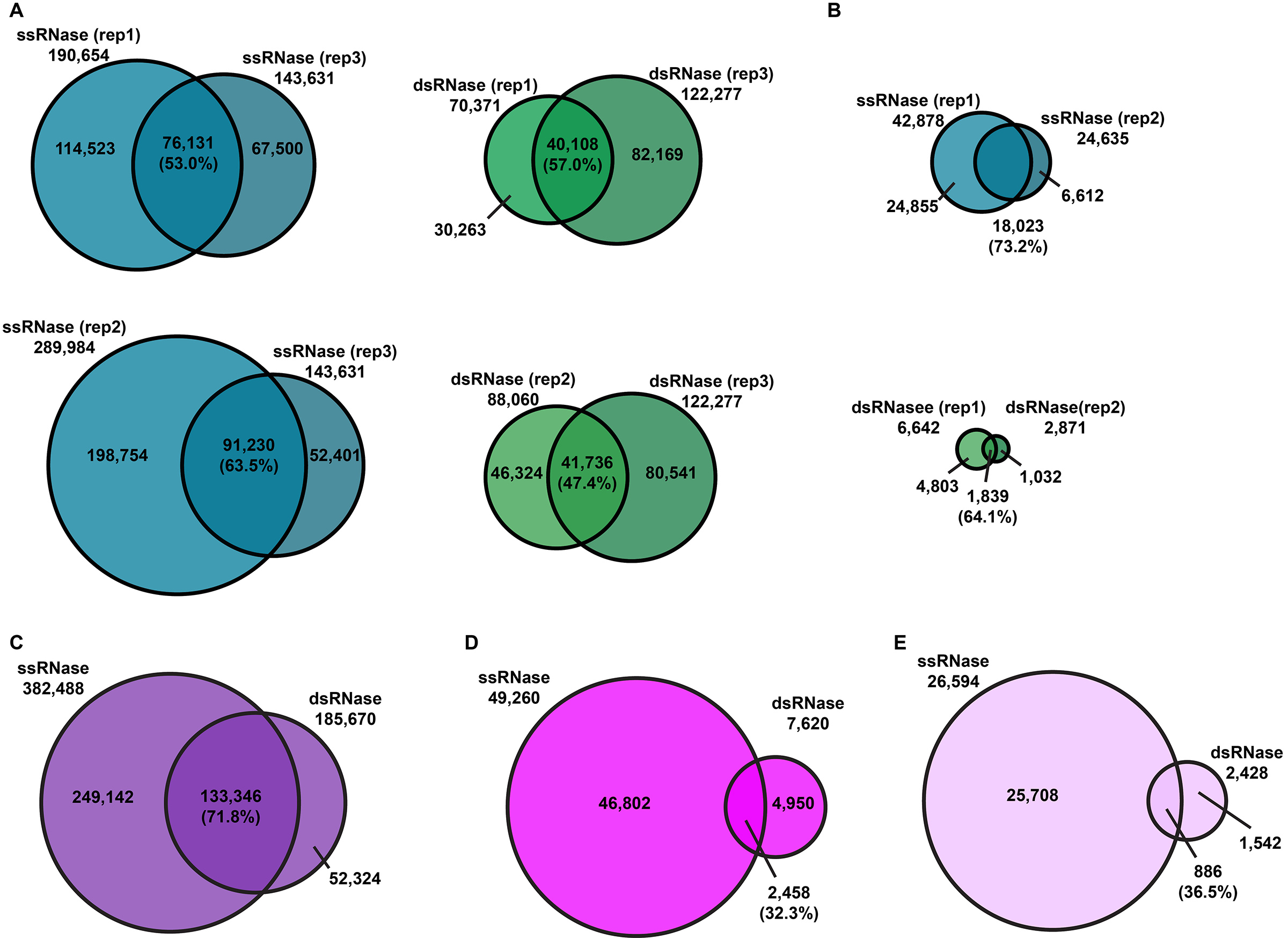

Supplement: Additional file 5 — PIP-seq is a reproducible approach (related to Figure 2). (A) Overlap in PPS calls between additional formaldehyde-cross-linked ssRNase-treated (blue) and dsRNase-treated (green) PIP-seq replicates. (B) Overlap in PPS calls between two replicates of UV-cross-linked ssRNase-treated (blue) and dsRNase-treated (green) PIP-seq replicates. (C) Overlap in PPS calls between formaldehyde-cross-linked ssRNase-treated and dsRNase-treated PIP-seq samples. (D – E) As (C), but for UV-cross-linked replicates (D) and the non-cross-linked experiment (E). [file gb-2014-15-1-r3-S5.jpeg]

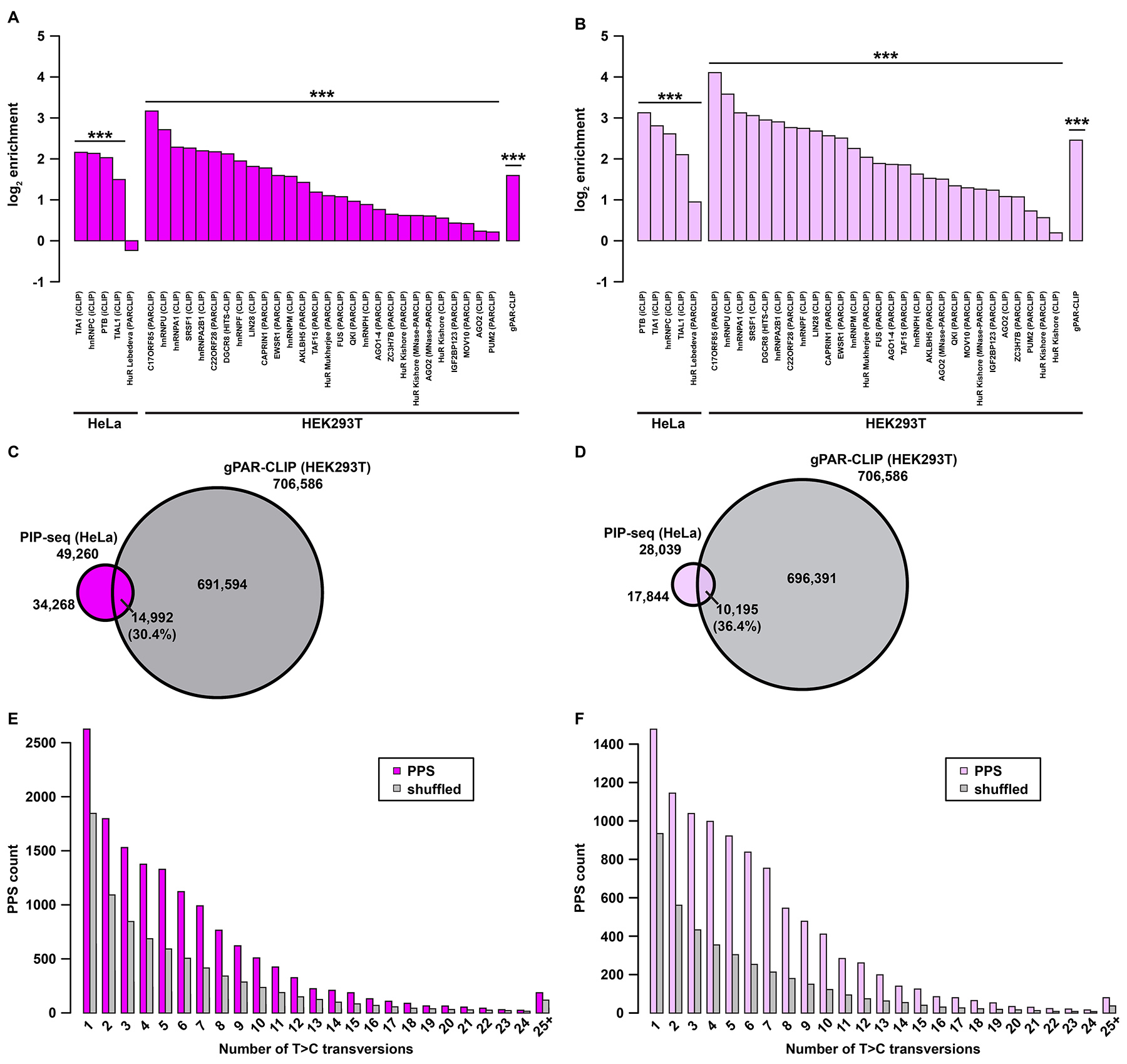

Supplement: Additional file 6 — Validation of PIP-seq by comparison with previously published RBP binding site datasets. (A) Overlap between PPSs identified from two UV-cross-linked PIP-seq samples and various CLIP datasets. Values are shown as log2 enrichment over shuffled background distributions. (B) As (A), but for a non-cross-linked PIP-seq sample. *** denotes P < 2.2 × 10^-16^ (chi-squared test). (C) Overlap between UV-cross-linked PPSs and 40-nucleotide T > C transversion event-containing loci from the gPAR-CLIP dataset (T > C transversion events less than 40 bp apart were merged to generate a dataset comparable to PPSs). (D) As (C), but for PPSs identified with no cross-linking. (E) Number of T > C transversion events per PPS identified using UV cross-linking (magenta) versus shuffled regions (gray). Values for the number of events per shuffled region are the average from ten random shuffles. (F) As (E), but for PPSs identified with no cross-linking. [file gb-2014-15-1-r3-S6.jpeg]

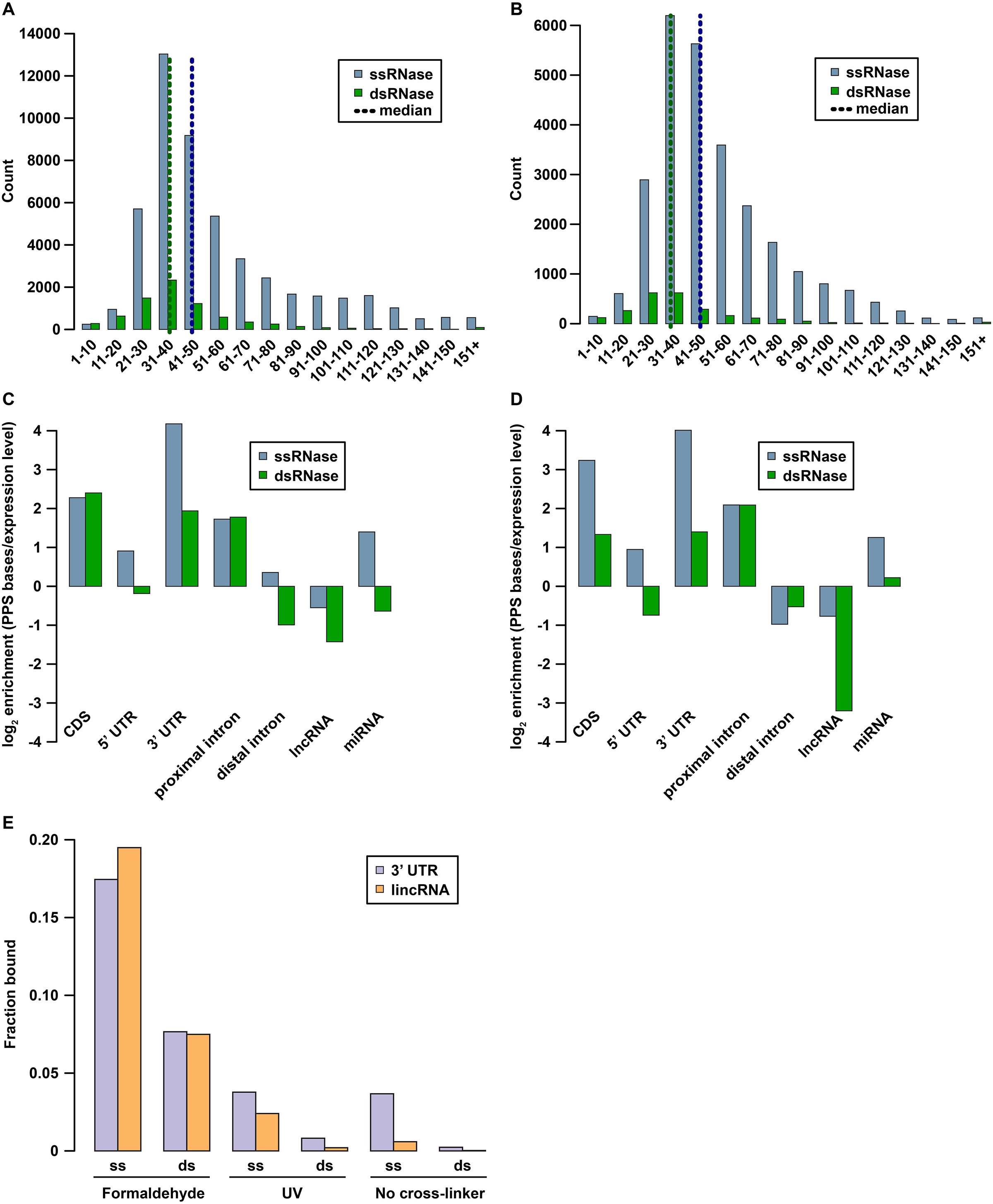

Supplement: Additional file 7 — Characterization of PPSs identified by UV-cross-linking and no-cross-linking PIP-seq experiments (related to Figure 3). (A) Distribution of ssRNase-treated (blue) and dsRNase-treated (green) PPS sizes from UV-cross-linked samples. Dashed lines represent mean PPS sizes (ssRNase, blue line and dsRNase, green line). (B) As (A), but for non-cross-linked PPSs. (C) Genomic distribution of UV-cross-linked PPS density, measured as PPS base coverage normalized to RNase digestion control read counts per genomic region. Proximal intron refers to 500 nucleotides at the 5′ and 3′ ends of introns. (D) As (C), but for non-cross-linked PPSs. (E) Fraction of base pairs covered by PPSs in 100 most highly expressed lncRNAs (orange bars) and expression-matched control mRNA 3′ UTRs (purple bars) for PIP-seq libraries made with ssRNase (ss) or dsRNase (ds) under the three different cross-linking conditions (as specified). [file gb-2014-15-1-r3-S7.jpeg]

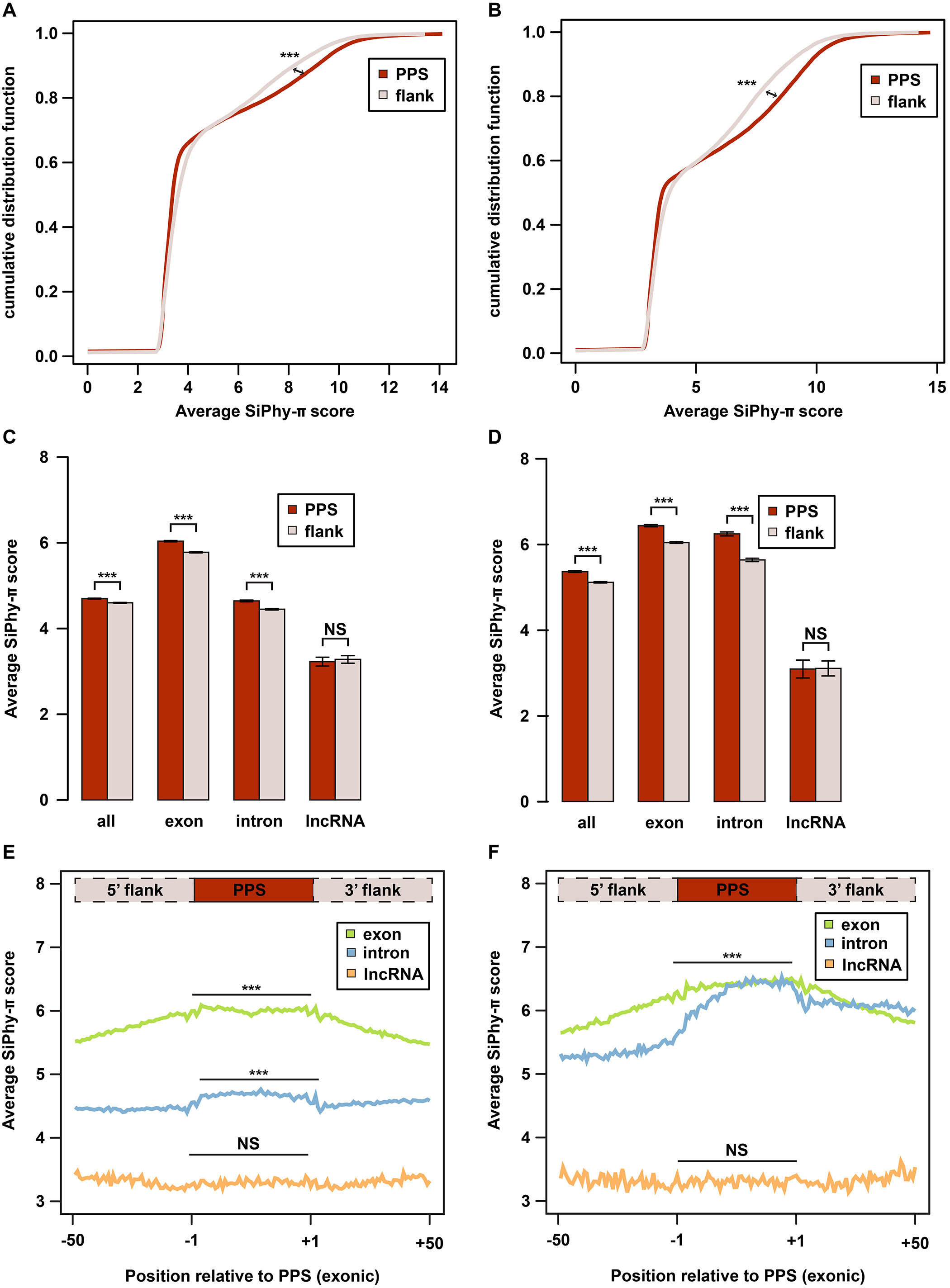

Supplement: Additional file 8 — PPSs identified by UV-cross-linking and no-cross-linking PIP-seq experiments are evolutionarily conserved (related to Figure 3). (A,B) Cumulative distribution of average SiPhy-π scores in PPSs identified by UV cross-linking (A) and no cross-linking (B). PPSs (red) are compared to flanking sequences (gray). (C,D) Comparison of average SiPhy-π scores between PPSs identified by UV cross-linking (C) and no cross-linking (D). PPSs (red) are compared to flanking sequences (gray) for various genomic regions. (E,F) Average SiPhy-π score profiles across the first and last 25 nucleotides of PPSs identified by UV cross-linking (E) and no cross-linking (F), as well as 50 nucleotides upstream and downstream of exonic (green line), intronic (blue line) and lncRNA (orange line) PPSs. *** denotes P < 2.2 × 10^–16^ (chi-squared test). NS, not significant. [file gb-2014-15-1-r3-S8.jpeg]

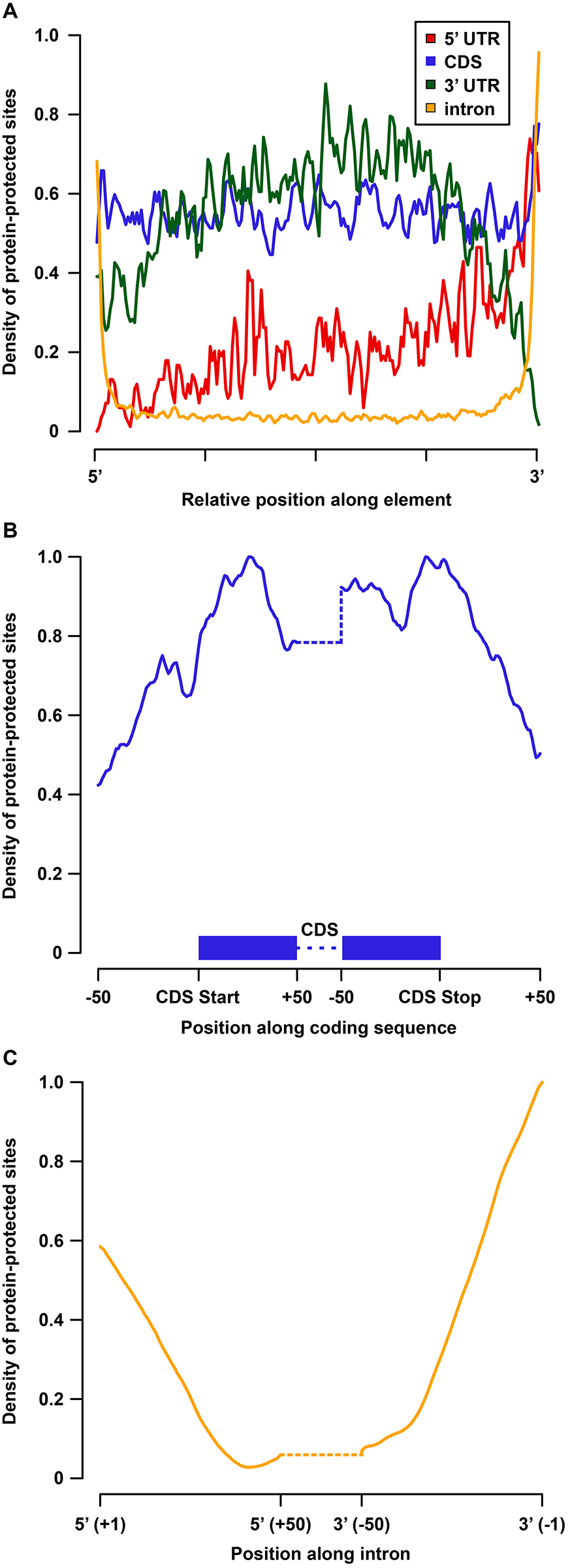

Supplement: Additional file 9 — Genomic distribution of PPSs identified by UV-cross-linking and no-cross-linking PIP-seq experiments (related to Figure 4). (A) Average PPS density for no-cross-linking PIP-seq across 100 equally spaced bins in various genic regions. Values are normalized separately for each genic region (for example, intron). (B) Average PPS density for no-cross-linking PIP-seq within 50 nucleotides of CDS ends. (C) Average PPS density for no-cross-linking PIP-seq within the first and last 50 nucleotides of introns. Dotted lines in (B,C) represent the remaining (unanalyzed) length of each element. [file gb-2014-15-1-r3-S9.jpeg]
